# An Automated System for Rapid Non-Destructive Enumeration of Growing Microbes

**DOI:** 10.1371/journal.pone.0008609

**Published:** 2010-01-07

**Authors:** Roanna London, Julie Schwedock, Andrew Sage, Heather Valley, Jamie Meadows, Michael Waddington, Don Straus

**Affiliations:** 1 Rapid Micro Biosystems, Inc., Bedford, Massachusetts, United States of America; 2 Accugenix, Inc., Newark, Delaware, United States of America; Massachusetts General Hospital, United States of America

## Abstract

**Background:**

The power and simplicity of visual colony counting have made it the mainstay of microbiological analysis for more than 130 years. A disadvantage of the method is the long time required to generate visible colonies from cells in a sample. New rapid testing technologies generally have failed to maintain one or more of the major advantages of culture-based methods.

**Principal Findings:**

We present a new technology and platform that uses digital imaging of cellular autofluorescence to detect and enumerate growing microcolonies many generations before they become visible to the eye. The data presented demonstrate that the method preserves the viability of the microcolonies it detects, thus enabling generation of pure cultures for microbial identification. While visual colony counting detects *Escherichia coli* colonies containing about 5×10^6^ cells, the new imaging method detects *E. coli* microcolonies when they contain about 120 cells and microcolonies of the yeast *Candida albicans* when they contain only about 12 cells. We demonstrate that digital imaging of microcolony autofluorescence detects a broad spectrum of prokaryotic and eukaryotic microbes and present a model for predicting the time to detection for individual strains. Results from the analysis of environmental samples from pharmaceutical manufacturing plants containing a mixture of unidentified microbes demonstrate the method's improved test turnaround times.

**Conclusion:**

This work demonstrates a new technology and automated platform that substantially shortens test times while maintaining key advantages of the current methods.

## Introduction

Since the 19^th^ century, counting the visible microbial colonies grown on semi-solid agar-based growth medium has been the dominant method for quantitative microbiological analysis. A key advantage of visual plate counting is its sensitivity for detecting growing cells; a visible colony will develop even when there is only a single culturable cell in a sample. The method detects a broad spectrum of prokaryotic and eukaryotic microbes and can simultaneously detect diverse species in a single sample. Plate counting methods can accommodate large volume liquid samples when combined with liquid filtration through membranes. The recovered microbial colonies are themselves essential for subsequent analyses since they contain populations of identical cells which are required as input for microbial identification, antibiotic susceptibility testing, biochemical analysis, and genetic characterization. Finally, visual plate counting owes its 130 years of dominance in large part to cost effectiveness and ease of use.

A major drawback of microbial culture methods is the length of time required for microbes to generate enough daughter cells to give rise to visible colonies. Testing times are particularly lengthy for slow-growing strains. While it is well known that in clinical applications long testing times can delay initiation of appropriate antimicrobial medical therapy, slow testing also has a high cost in pharmaceutical and personal care product manufacturing.

The need for faster microbial enumeration results has spurred technology development for almost a century. For example, as early as 1920, Frost developed a method for rapidly detecting microbial growth by microscopic detection of nascent microcolonies [1]. In contrast to visual plate counting, microscopic examination is impractical when the microbial count is low. New technologies for rapid microbial detection – including nucleic acid, bioluminescent, and immunoassay-based methods – have had a substantial impact in clinical diagnostics and food pathogen testing. The new methods have had less impact in pharmaceutical and personal care product manufacturing which are more closely tethered to culture methods because of regulatory requirements.

Several factors have stymied extensive implementation of new technologies in the pharmaceutical and personal care product industries. Regulations require that manufacturers must validate the new method by demonstrating its equivalence to the gold standard culture method [2], [3]. However, demonstrating equivalence is difficult for new technologies that use principles distinct from the culture method. For example, it is difficult to compare the microbial colony count from a water sample that harbors a complex microbial population to the results of an ATP-bioluminescence technology that delivers results in relative light units. Furthermore, most new methods kill the microbes precluding growth of the pure cultures that are required as input for today's microbial identification methods. Identifying microbes is an essential tool for investigating the source of contamination events and for maintaining control of the manufacturing environment [4]. Some methods can only address a small range of microbiological quality control applications. For example, flow cytometric methods [5] only operate on small liquid samples (generally less than about 0.5 ml), and cannot test air, surface, or large volume liquid samples which are critical in manufacturing quality control. Methods that do not rely on cell replication, such as solid phase cytometry [6], complicate demonstration of equivalence to the current method due to the preponderance of viable but non-culturable microorganisms in the environment [7]. Hence these methods can deliver enumeration results that are substantially higher than the standard plate counting methods. Finally, some methods for rapid microbial enumeration require more hands-on labor than does the plate count method.

Our goal in developing the technology described here is to provide a microbial enumeration test that is faster than visual plate counting, but maintains the advantages of the traditional method. We reasoned that a method based on the same principles as traditional plate counting would facilitate regulatory validation, give equivalent counts, address the same broad spectrum of applications as traditional plate counting, and be compatible with microbial identification technology.

**Figure 1:**
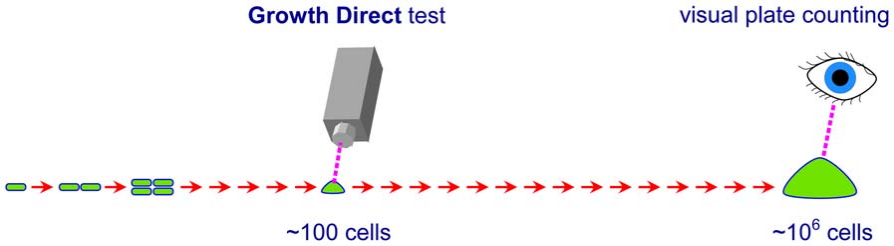
Digital imaging of cellular autofluorescence detects microcolonies generations before they become visible to the eye.

The Growth Direct™ System that we describe counts colonies growing on the same nutrient media as does the traditional method. The new method, however, detects the colonies many generations before they become visible to the eye by using digital imaging to detect the cellular autofluorescence of the growing colonies (Figure 1). Thus, the technology retains the advantages of traditional culture methods while reducing long test turnaround times. The system's automation eliminates tedious manual counting and decreases the chance for human error.

The technology exploits the fact that all types of cells fluoresce in the yellow-green when illuminated with blue light [8]. Studies using various cell types have implicated oxidized flavins (riboflavin, FAD, FMN) as the source of much of the signal in this spectral range [9]–[12]. As early as 1959, Chance and Thorell reported microscopic detection of single autofluorescent yeast cells [13]. More recently, the design of a handheld system for detecting microbial autofluorescence was described [14].

We describe the new methodology and show that it non-destructively detects growing microcolonies much sooner than they become visible to the naked eye. We also present feasibility studies for key pharmaceutical quality control applications using the Growth Direct System.

## Results

### Overview of the Detection Technology

**Figure 2:**
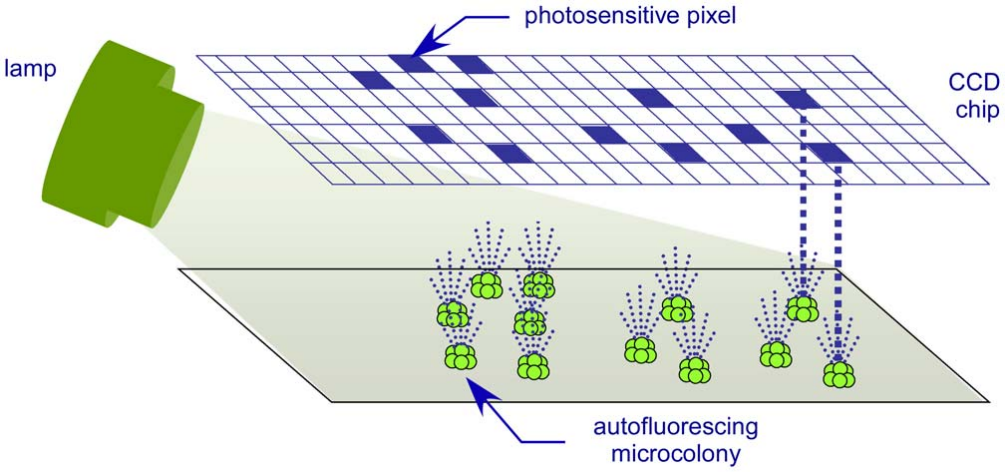
The Growth Direct System detects microcolony autofluorescence.

The Growth Direct System detects microcolonies by illuminating them with blue light and directing the resulting cellular autofluorescence, without magnification, onto a CCD chip – an array of independent photosensitive pixel elements. Image analysis software automatically counts the clusters of illuminated pixels overlying each microcolony (Figure 2).

**Figure 3:**
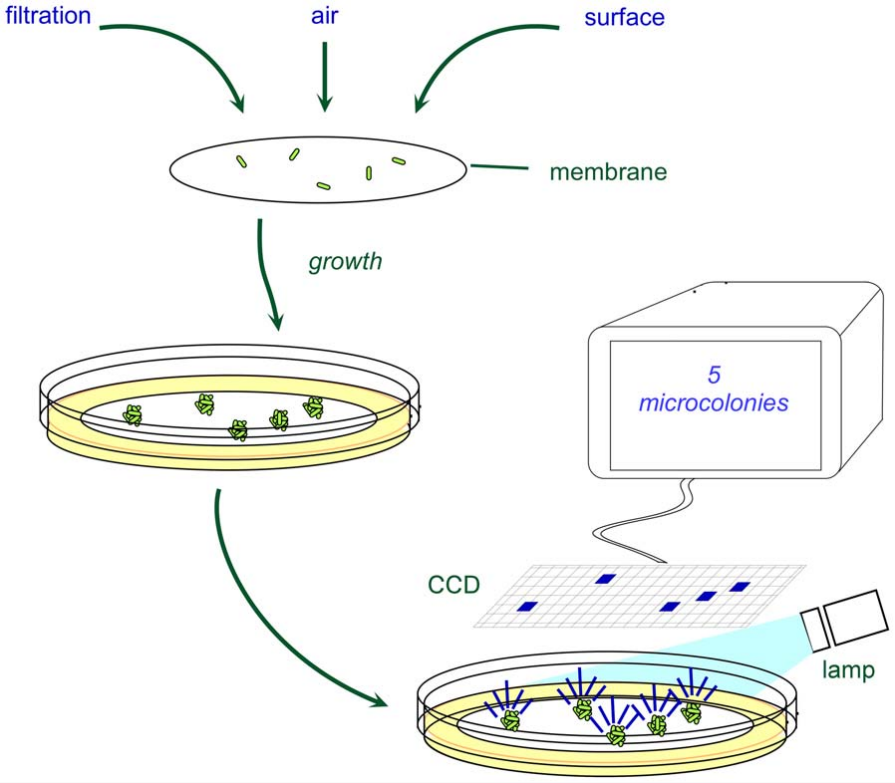
The workflow of the Growth Direct System is similar to that of visual plate counting. For clarity, the diagram does not show the system's optical or mechanical components.

The work flow of the Growth Direct method is essentially identical to that of traditional membrane filtration-based visual plate counting assays (Figure 3). Microbes are filtered onto a membrane which is then placed on nutrient agar medium in a Growth Cassette™ (Figure S1), incubated for a substantially shorter time than the traditional test, and automatically counted.

The Growth Cassette (Figure S1) has several properties that facilitate imaging. Its lid is made from low-fluorescence plastics and includes a clear optical window. To enable in-focus images from the entire cassette, the agar is poured against a removable pouring lid with a flat surface, eliminating the usual meniscus from the pouring surface. As for traditional microbial enumeration of large volume liquid samples by membrane filtration, microbes are grown on membranes placed on the agar surface. The membrane filters are composed of mixed esters of cellulose, the same material used for the traditional method, and are dyed black to minimize fluorescence background.

Figures S2 and S3 show the functional organization of the Growth Direct System. The microbiologist introduces carriers that hold up to 20 Growth Cassettes at a loading station with room for 8 carriers. The Growth Cassettes are robotically transferred from the carrier to one of two independently controlled incubators which together have a capacity of 320 Growth Cassettes. For imaging, cassettes are automatically removed from the incubator, imaged using the CCD camera, and returned to the incubator. When the test is complete the cassettes can be either returned to a carrier or automatically discarded as waste at the microbiologist's discretion. A simple user interface allows the microbiologist to track the cassettes in the system and monitor the numerical results.

**Figure 4:**
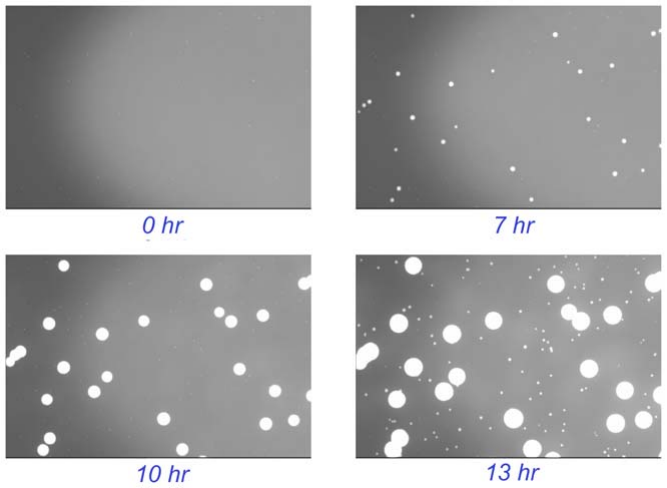
The Growth Direct System distinguishes growing microcolonies from fluorescent debris by analyzing multiple images over time. The figure shows a time series of growing autofluorescent *E. coli* and *B. cepacia* microcolonies.

**Figure 5:**
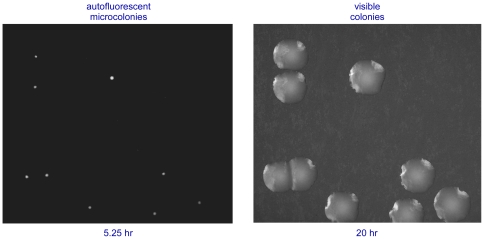
Comparing the number and pattern of microcolonies detected by the Growth Direct System to visible colonies. *E. coli* microcolonies detected by the Growth Direct System are shown in the left panel and the visible colonies to which they give rise are in the right panel. The number and pattern of microcolonies are identical in each panel.

To accurately distinguish growing microcolonies from fluorescent debris, the Growth Direct System monitors the increase in the growing microcolonies' autofluorescent signal over time (Figure 4). At specified time intervals the Growth Cassettes are automatically removed from the incubator, imaged, and then returned to the incubator until the next imaging time point. In this way, the system acquires an image time series for each sample. Equivalence of the rapid method to the traditional plate technique can be demonstrated by comparing the pattern of microcolony autofluorescence to the visible colonies that eventually appear (Figure 5). The figure also illustrates a key feature of the technology. Because the method can detect and enumerate microcolonies without harming them, subsequent incubation generates visible colonies that can be used for microbial identification. We demonstrated that the system's automated image analysis software accurately detects growing microcolonies (Figure S4) including those with complex morphologies (Figure S5).

### Choosing a Spectral Regime for Detecting a Broad Range of Microbes Using Autofluorescence

To achieve early detection for the broad range of microbial species, we explored the optimal autofluorescent response in the visible spectrum for 47 microbes representing breadth in phylogeny, metabolism, developmental program, and pigmentation (Table 1). By scanning lawns of microbes grown on membrane filters, we determined the relative microbial autofluorescence intensity at excitation and emission wavelengths covering much of the visible spectrum.

**Table 1:** Strains analyzed by two-dimensional fluorescence spectroscopy.

**Bacteria**		
*Bacillus cereus*	*Geobacillus stearothermophilus*	*Pseudomonas putida*
*Bacillus subtilis*	*Kocuria rhizophila*	*Pseudomonas stutzeri*
*Bacteroides fragilis*	*Methylobacterium extorquens*	*Rhodococcus erythropolis*
*Brevundimonas diminuta*	*Methylobacterium radiotolerans*	*Roseomonas gilardii*
*Burkholderia cepacia*	*Micrococcus luteus*	*Salmonella enterica*
*Chromobacterium violaceum*	*Myxococcus xanthus*	*Serratia marcesens*
*Clostridium sporogenes*	*Porphyromonas gingivalis*	*Staphylococcus aureus*
*Corynebacterium pseudodiphtheriticum*	*Prevotella melaninogenica*	*Staphylococcus epidermidis*
*Corynebacterium xerosis*	*Propionibacterium acnes*	*Staphylococcus warneri*
*Deinococcus proteolyticus*	*Pseudomonas aeruginosa*	*Streptomyces coelicolor*
*Escherichia coli*	*Pseudomonas fluorescens*	*Vibrio natriegens*
**Yeast and molds**		
*Aspergillus flavus*	*Candida albicans*	*Saccharomyces cerevisiae*
*Aspergillus fumigatus*	*Candida parapsilosis*	*Schizosaccharomyces pombe*
*Aspergillus niger*	*Penicillium camemberti*	*Sporotrichum pruinosum*
*Aspergillus versicolor*	*Penicillium notatum*	*Zygosaccharomyces rouxii*
*Aureobasidium pullulans*	*Penicillium roquefortii*	

**Figure 6:**
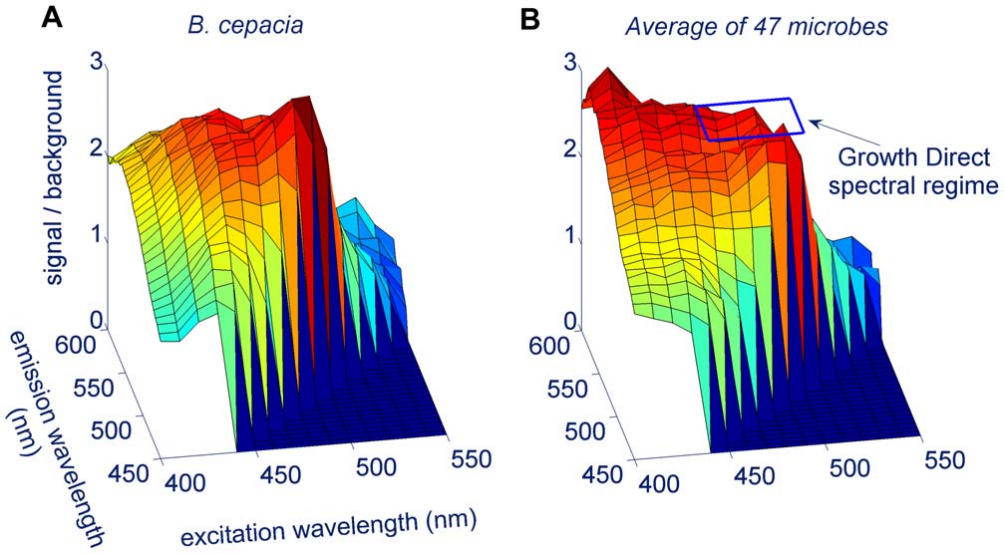
The microbial autofluorescence spectral response. The plots show the ratio of signal to background for two dimensional fluorescence scans of (A) *B. cepacia* cells and (B) the 47 microbes listed in Table 1 (averaged). The spectral regime used by the Growth Direct System is indicated (excitation: 450–500 nm, emission 510–560 nm).

Figure 6A plots the ratio of the microbial autofluorescent signal to the background for one representative strain and medium combination (*Burkholderia cepacia* growing on TSA medium) as a function of excitation and emission wavelength. Figure 6B plots the average signal to background levels for 47 species in Table 1. The ideal spectral regime should deliver high signal to background across a broad range of microbes and should minimize the potential for introducing cellular damage by avoiding high energy (short wavelength) irradiation. Based on these criteria, we chose a spectral regime of 450 to 500 nm for excitation and 510 to 560 nm for emission.

The robustness of the chosen spectral regime is supported by the fact that the Growth Direct System has detected many diverse strains from culture collections and environmental samples (Table S1). The list includes both eukaryotic (fungal) and the bacterial strains. The system detects microbes from disparate bacterial phyla including Proteobacteria (including the alpha, beta, gamma, and delta subdivisions), Gram positive bacteria (both high and low GC content, including mycoplasmas), Flavobacteria and Deinococci. Among the fungi, we showed that the system detects strains from the Zygomycota, Ascomycota, Basidiomycota, and the Deuteromycota groups. While the organisms we tested do not cover the whole of phylogenetic diversity, they do cover the diversity that would be detected by current tests in a manufacturing environment. These organisms also cover a wide range of morphological diversity, including species that make dark pigments (*Chromobacterium violaceum*, *Aureobasidium pullulans*), those that make fluorescent pigments (*Pseudomonas fluorescens*), molds with compact colony structures (*Aspergillus niger*, *Penicillium notatum*), and molds with spreading growth patterns (*Alternaria alternata*, *Trichoderma asperellum*). In addition, we have tested hundreds of water, air, and surface samples which gave rise to thousands of visible colonies. To date, every isolate that gave rise to a visible colony was also detected by the system using cellular autofluorescence. Therefore we conclude that the chosen spectral regime detects the same broad range of replicating microbes as the traditional visual plate counting method.

### Demonstrating that the Spectral Regime Does Not Harm Microorganisms

We performed experiments to determine whether being processed through the Growth Direct System could harm cells due to light or heat-induced injury. Some microbes are more sensitive to light than others, but it would be impossible to test all organisms. Therefore to test whether the system is destructive, we chose an “over-stress” strategy in which we subjected well characterized test strains to conditions that are substantially more extreme than would be experienced in the system.

**Figure 7:**
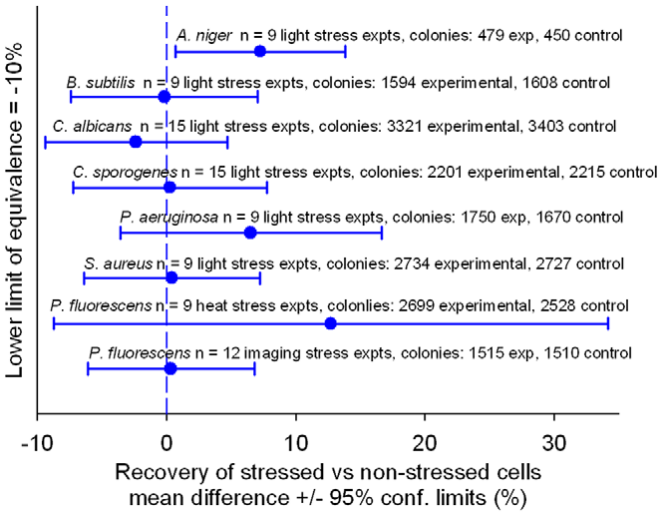
Demonstrating the method is non-destructive. To show that the light and heat stress occurring during processing on the Growth Direct System do not harm cells, we tested the ability of standard test strains to withstand stresses more extreme than those that would normally be experienced on the Growth Direct System. To test the effect of light stress, microbes were exposed to 10 times the level of blue light that they would actually experience on the system (top six lines). To test the effect of heat stress, *P. fluorescens* was subjected to an imaging cycle at an elevated temperature (second from the bottom) and to an accelerated imaging regime which caused heat and imaging cycles to occur at twice the normal frequency (bottom line). To test the equivalence in microbial recovery for samples with and without stress, we used the TOST method. The figure plots the percent difference of the mean titer of the stressed samples vs. the untreated controls (•) and indicates the 95% confidence intervals. The number of replicates for each experiment and the total number of colonies analyzed are indicated above each line.

To test for sensitivity to illumination by the system's blue light, we irradiated freshly plated cells with ten times the fluence used in the system. We used the same illumination instrumentation as is used in the Growth Direct System and made sure that the microbes were not exposed to harmfully high temperatures as a result of photonic heating by the intense light. Figure 7 details the recovery of irradiated vs. non-irradiated cells of six standard test strains used in the pharmaceutical industry. The results show that the recovery of irradiated cells is statistically equivalent to or better than those of non-irradiated cells, at the 95% confidence level.

The temperature of the Growth Cassettes briefly increases during the illumination step and also during a pre-imaging condensation removal step in which the optical lid is bathed in warm air. To test whether heating by the system could diminish the recovery of environmental microbes that are temperature sensitive, we measured the recovery of a known heat sensitive microbe, *P. fluorescens*. *P. fluorescens* does not grow when incubated at 42°C and its growth is measurably slowed at 35°C (Figure S6). We exposed this organism to the condensation removal/imaging cycle every 1.5 hours, which is is twice the frequency of imaging cycles that a cassette would experience in the system. Figure 7 shows that the recovery of this strain after exposure to this rapid cycling regime was equivalent to that of the unheated, non-illuminated controls. We conclude that there is no decrease in recovery of *P. fluorescens* due to heating during processing by the Growth Direct System.

### Range, Linearity, and Precision

**Figure 8:**
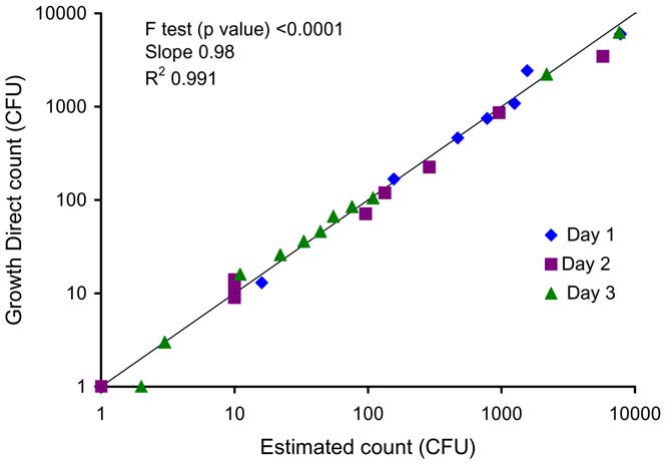
Range, linearity, and correlation to visual plate counting. The plot correlates the results obtained using the Growth Direct System to the visual plate counting method for samples containing varying numbers of *E. coli* CFU. For this blinded experiment, samples were prepared and analyzed either using the Growth Direct System or visual plate counting. The Growth Direct System results were determined after incubating for 5 hrs by one analyst. Visual plate counting results were determined after overnight growth (≥18 hr) by a second analyst.

Figure 8 shows that the method is accurate and linear for *Escherichia coli* up to about 10,000 CFU/ml – which is about 30 times higher than the limit of the visual plate counting method (generally about 300 CFU/ml for bacterial colonies). This increase in range is due to the small size of the detected microcolonies. Enumerating small microcolonies allows for much higher colony densities to be reached before colony overlap begins to impede accuracy. We also showed that the system has the potential to dramatically extend the linear range to more than 1×10^6^ CFU/ml due to the linear increase in autofluorescence with cell number even on confluent lawns microbes with no distinct microcolonies (Figure S7). We also determined the precision of the system by counting 20 replicates of *B. cepacia* at low and high concentrations. The coefficient of variation was 12% for samples containing around 150 CFU and 5.2% for samples containing around 1500 CFU. The results show that the system's precision is well within the acceptable limit of <15% for microbiological quality control tests [15].

### Determining the Time Saved by Autofluorescent Detection of Microcolonies

To measure the system's ability to accelerate microbial detection, we determined the times at which it first can detect representative microbes of varying cell size. We compared the time to detection of autofluorescent microcolonies of *E. coli*, *Brevundimonas diminuta*, and *C. albicans* to the time required to detect colonies by eye (Table 2). The table compares the median time at which the autofluorescent microcolonies are first detected to the time required for the colonies to grow to be just visible to the naked eye (0.5 mm in diameter). The data show that the rapid method first detects these organisms 55% to 90% sooner than they can be detected by eye.

**Table 2:** Time savings achieved for model microbes using autofluorescent detection.

	Time to detection (hr)	
	autofluorescence^1^^,^ 2	visual^3^
***C. albicans***	2.2±0.2	22
***E. coli***	3.1±0.1	8.5
***B. diminuta***	9.8±0.2	22

1 Time at which 50% of the autofluorescent microcolonies were first detected by prototype software.

2 Ranges represent the 95% confidence interval.

3 Time at which colonies become visible to the naked eye (0.5 mm in diameter).

### Determining the Number of Cells in the Earliest Detectable Microcolonies

**Figure 9:**
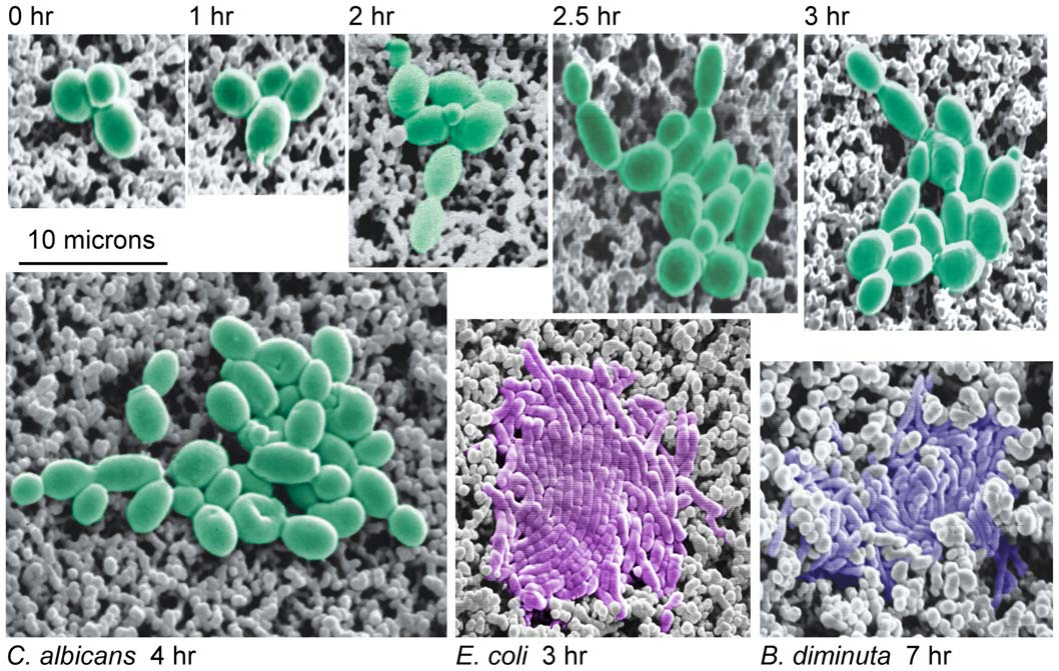
Scanning electron microscopic images of microcolonies on membranes. The microcolonies shown in false color are composed of cells of *C. albicans* (green), *E.coli* (violet), and *B. diminuta* (blue). *C. albicans* colonies are first detected by the Growth Direct System between 2 and 2.5 hours, while *E. coli* colonies are first detected at around 3 hours. *B. diminuta* is detected at about 10 hours (colony shown is a 7 hour colony).

We determined the number of cells in the earliest detectable *E. coli*, *B. diminuta* and *C. albicans* microcolonies using scanning electron microscopy (SEM). Figure 9 shows representative SEM images of early *E. coli*, *B. diminuta* and *C. albicans* microcolonies growing on membranes at various times after plating. The results of the SEM study indicate that the system first detects autofluorescent microcolonies of *E. coli*, *C. albicans*, and *B. diminuta* when they contain about 120, 12, and 980 cells respectively (Table 3).

**Table 3:** The relationship between cell volume, intrinsic fluorescence, and number of cells at the time of detection for model organisms.

	Cell volume^1^^,^ 2 (cubic microns)	Relative volume per cell	Fluorescein equivalents per cell^2^^,^ 3 (*F_cell_*)	Relative fluorescence per cell	Number of cells in microcolony (SEM^2^) (*N_thresh_*)	Number of cells in microcolony (wash-off^2^) (*N_thresh_*)	Fluorescein equivalents at time to detection^2^^,^ 4 ^,^ 5 (*F_thresh_*)
*C albicans*	49±5	5.6	3×10^3^±1×10^3^	5.4	12±2	8.0±0.8	3.8×10^4^±1.5×10^4^
*E. coli*	8.8±0.7	1	5.6×10^2^±1.4×10^2^	1	1.2×10^2^±0.2×10^2^	1.7×10^2^±0.3×10^2^	6.6×10^4^±3.2×10^4^
*B. diminuta*	2.1±0.4	0.23	81±7	0.14	9.8×10^2^±2.9×10^2^	ND	7.9×10^4^±2.6×10^4^

1 volume estimates are based on length measurements made from calibrated light microscopy images.

2 ranges represent 95% confidence interval.

3 as measured on the Growth Direct System with calibrated beads.

4 based on Scanning Electron Microscopy estimate of cell number.

5 See Table 2 for time to detection.

For *E. coli* and *C. albicans* we also used a microbiological method to determine the number of cells in the microcolonies. We incubated parallel samples for various times before washing the cells off of the membranes and enumerating the CFUs by visual plate counting. By immersing and agitating the membrane we were able to recover >90% of the *E. coli* and *C. albicans* cells (but not *B. diminuta*) from the membrane. We estimated the number of cells in microcolonies at various times by dividing the number of original colonies by the number of colonies generated from the cells washed-off of the membrane (see materials and methods). Using this method we found that at the time that the system first detects *E. coli*, and *C. albicans*, microcolonies contain about 170 and 8 cells respectively, which compares well to the results obtained using the SEM approach (Table 3).

Using the wash-off method described above, we determined that small (0.5 mm diameter) visible *E. coli* and *C. albicans* colonies contain about 5×10^6^ and 2×10^5^ cells respectively. Interestingly, our results show that the measured doubling time of these microbes on membranes is similar to the doubling time observed in aerated, log phase broth cultures. Analysis of this data also showed that the duration of the lag phase on the membranes was 40 min and 60 min for *E. coli* and *C. albicans*, respectively (Table S2).

### Microbial Autofluorescence Is Correlated to Cellular Volume

**Figure 10:**
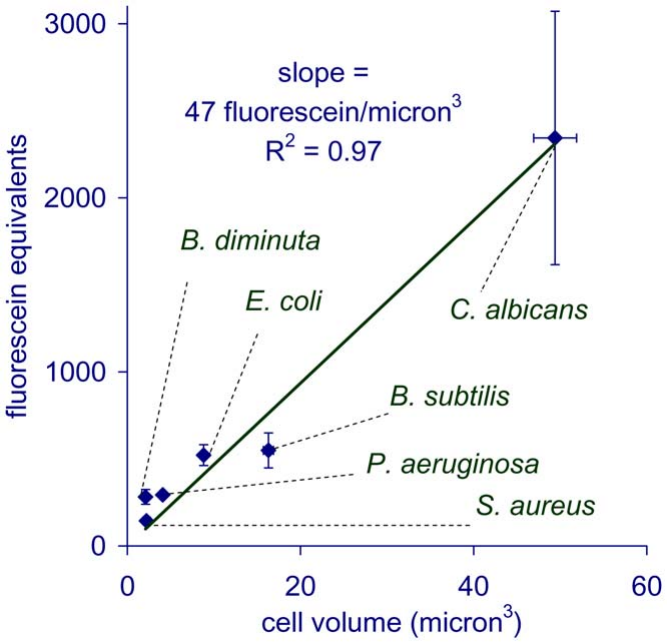
Intrinsic microbial cellular fluorescence is proportional to cellular volume. The data indicates that using the Growth Direct spectral regime, microbes fluoresce with an intensity equivalent to about 47 fluorescein molecules for every cubic micron of cellular volume.

To understand the variation in cellular autofluorescence across microbial strains we used flow cytometry to compare the cellular fluorescent response of various microbes to that of particles containing a known amount of fluorescein. We found that the intrinsic cellular fluorescence, ***F_cell_***, is correlated to cellular volume, ***V_cell_*** (Figure 10). The slope of a plot of ***F_cell_*** vs ***V_cell_*** indicates that each cubic micron of cellular volume contributes about 47±4.4 fluorescein equivalents (95% confidence limit) of autofluorescence.

### Relationship of a Strain's Intrinsic Cellular Fluorescence to the Number of Cells Required to Detect a Microcolony

Microcolonies of strains with high intrinsic cellular fluorescence should be detected when they have fewer cells than those with low cellular fluorescence. To quantify the relationship between cellular fluorescence and time to detection, we used the intrinsic fluorescence values determined by the Growth Direct System rather than those obtained using the flow cytometer, because the two systems have different optics and spectral regimes. We determined the relative cellular autofluorescence response to known numbers of cells applied to membranes for *E. coli*, *C. albicans*, and *B. diminuta*, the strains for which we had determined the number of cells at the earliest time to detection (Table 3). The mean cellular autofluorescence intensities for *C. albicans*, *E. coli*, and *B. diminuta* were about 3000, 560, and 80 fluorescein equivalents per cell respectively. As expected, the cellular autofluorescence responses using the Growth Direct System and flow cytometry have different absolute values for these strains but similar relative values.

A simple model predicts that the fluorescence of microcolonies at the threshold of detection (***F_thresh_***) should (1) be the same for different microbes and (2) equal the product of the intrinsic cellular fluorescence and the number of cells in the microcolonies. So,
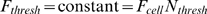
(1)where ***F_cell_*** is the intrinsic cellular fluorescence and ***N_thresh_*** is the number of cells in the earliest detectable microcolonies. This simple model predicts that number of cells in the earliest detectable microcolonies should be inversely proportional to the intrinsic cellular fluorescence. We found that this prediction was borne out when we plotted the values of ***N_thresh_*** versus 1/***F_cell_*** for *E. coli*, *C. albicans*, and *B. diminuta* (Figure S8). The value of ***F_thresh_***, estimated from the slope of the best fit line indicates that the system first detects microcolonies when they reach a threshold fluorescence level equivalent to about 81,000 fluorescein equivalents. Using this estimate, Equation (1), and the result 1/***F_cell_*** = *47* ***V_cell_*** fluorescein equivalents this simple model predicts the number of cells required to detect a microcolony as a function of cell volume, ***V_cell_***,:
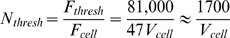
(2)Note that this simplified model does not take into consideration complicating factors that may pertain for certain microbes such as cellular “burrowing” under the surface of the membrane and secondary compounds that either enhance or quench fluorescence.

### A Model for Estimating Time to Detection Based on Key Parameters

Assuming the simple model described above, the time at which microbes are first detected by the Growth Direct System can be modeled as a function of four microbe-specific parameters: the time that elapses before cells begin dividing (the pre-growth time, which includes injury recovery, germination, and/or lag phase), the doubling time, the cell volume, and number of cells per CFU. The expected relationship is described in Equations 3 and 4:
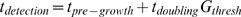
(3)where, ***t_detection_*** is the time to detection; ***t_pre-growth_*** is the pre-growth time; ***t_doubling_*** is the doubling time. Using Equation (2), ***G*** *_t_*_***hresh***_, the number of generations needed to form detectable microcolonies can be expressed as a function of the number of cells per CFU, ***n_CFU_***, and ***V_cell_***.
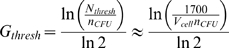
(4)


### Predicting the Time Savings for Samples with Mixed Populations of Microbes

**Figure 11:**
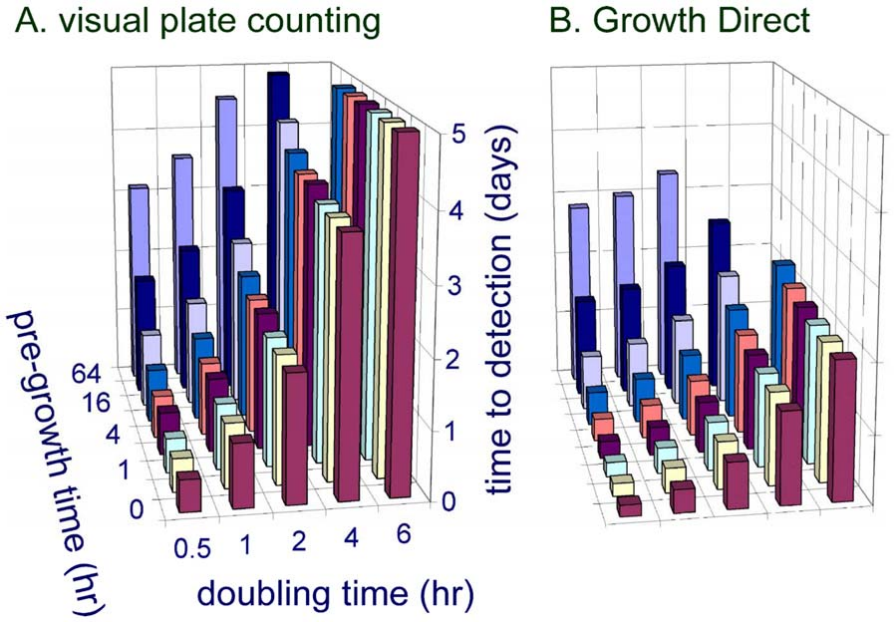
Predicted time savings using digital imaging of cellular autofluorescence. The predicted times required to detect colonies are shown in panel A (visual detection) and panel B (imaging cellular fluorescence). The calculated detection time for a colony – for either the traditional or rapid method – depends on the time before growth begins (pre-growth time) and the doubling times of the microbe (see model in text). For simplicity, calculated results are shown only for microbes with threshold detection parameters similar to those of *E. coli*. However, these results should also apply to the substantial fraction of strains that have similar colony morphologies and cellular autofluorescence levels. The calculations also make the simplifying assumption that colonies originate from single cells.

**Figure 12:**
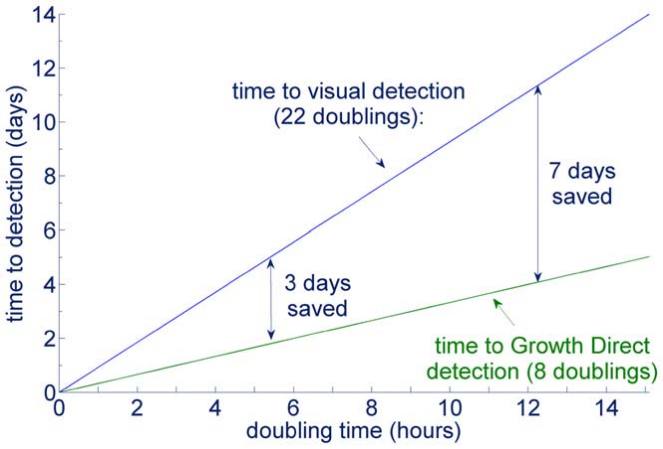
Relation of doubling time and time savings using the Growth Direct System. For two microbial strains that are otherwise similar, the Growth Direct System provides the largest time savings for the more slowly growing strain.

Pharmaceutical manufacturing quality control samples can harbor complex populations of diverse microbes requiring a range of incubation times to grow into visible colonies. In general, culture test duration is determined by the microbes of interest that are the slowest to appear in a given test application. For example, in pharmaceutical manufacturing quality control, yeast and mold tests are typically run for 5–7 days, product bioburden tests for 2–5 days, water tests for 3–7 days, air and surface environmental monitoring tests for 2–7 days, and finished product sterility tests for 14 days. Given knowledge about the number of cells required to detect colonies either visually or by using autofluorescence and the number of cells per CFU, it is possible to predict the times for autofluorescent or visual detection as a function of pre-growth time and doubling time using Equations (3) and (4). Figure 11A plots the visual plate counting time to detection as a function of pre-growth and doubling time for microbes that would be detected in a 5 day test, assuming the number of cells required for visual detection is 5×10^6^, the same as for *E. coli*. Figure 11B shows the predicted time to detection for the same microbes, those that could be detected visually in 5 days, when using the Growth Direct System, making the assumption that, like *E. coli*, the colonies can be first detected at the 128-cell stage. The model predicts the largest time savings for the most slowly growing microbes (Figure 12). This follows from the fact that the Growth Direct System detects microcolonies about 15 generations before they become visually detectable. The longer the generation time the more time is saved using the rapid method. The time savings relative to the traditional 5 day test are smaller for microbes with a combination of very long pre-growth times and rapid doubling times. Note that the theoretical treatment above applies to the initial detection of autofluorescent microcolonies. In practice, the system software requires that a putative fluorescent microcolony grows over multiple image time points before it is classified as a growing microcolony. Thus, the time at which the system reports detection of a microcolony is delayed relative to the initial detection by an amount of time that depends on the imaging interval (generally about 3 hours for pharmaceutical applications).

### Detecting Stressed and Injured Microbes

Microbes isolated in manufacturing plants may be injured or stressed by harsh environmental conditions (e.g., desiccation, temperature extremes, or nutritional stress) or treatment by disinfectants or heat. Injured or stressed microbes generally require time to recover or adapt before beginning to replicate on growth medium. To test the ability of the new method to provide time savings for detecting stressed or injured microbes we subjected the common water microbe, *R. pickettii*, to several treatments that are representative of those experienced by water isolates. Our results demonstrate that the time savings provided by the Growth Direct System compared to visual plate counting were similar for healthy log phase *R. pickettii* cells, cells subjected to prolonged incubation (10 days) in pure water at either 4°C or room temperature, and cells injured by chlorine treatment (Table S3). The results of this experiment are consistent with the expected result that the treated microbes have a more extended pre-growth time than the log phase cells followed by normal growth – regardless of which detection method is being used. Because the rapid and traditional methods experience the same extension of the pre-growth phase, the time savings delivered by the rapid method are similar whether the cells were treated or not. We have obtained similar results using other species treated with various disinfectants and high heat (data not shown).

### Rapid Detection of Microbes in Key Pharmaceutical Testing Applications

**Figure 13:**
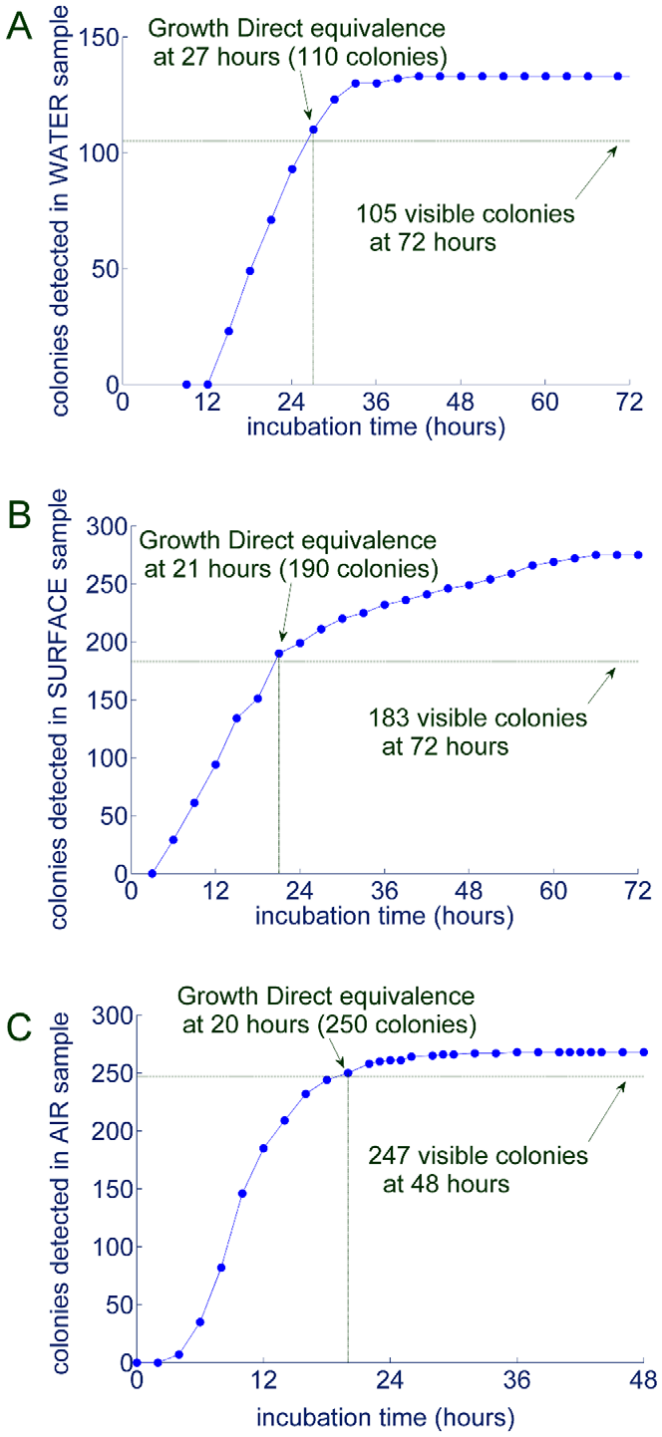
Rapid time to results for three key environmental microbiological testing applications. Samples from pharmaceutical facilities representing three common quality control applications were analyzed: (A) water, (B) surface, and (C) air. The figures plot the appearance of microcolonies over time (–•–). The visual plate count obtained after letting the colonies on the growth cassettes continue to grow for the traditional testing time is also indicated (−−−).

In pharmaceutical manufacturing, the majority of microbial enumeration test samples are filterable liquids or environmental (air and surface) samples. These samples generally contain mixed populations of unidentified environmental microbes. We tested representative samples of this type from pharmaceutical facilities to determine the feasibility for attaining substantial time savings using the new method compared to the traditional visual plate counting approach. We ran the tests for the full traditional testing time to determine the number of visible colonies that would be detected in the samples by visual plate counting. We then ascertained the time at which the system detected the number of colonies that later became visible on the same cassettes after the traditional testing time. Figure 13 shows the time course of the detection of microbes for water, air, and surface samples from pharmaceutical facilities using the commercial system's fully automated sample processing and analysis. The plots indicate the time at which the Growth Direct System detects the same number of colonies that were later detected at the end of the traditional testing time period. For this water sample, the Growth Direct System saved almost 2 days compared to the traditional 3 day test (Figure 13A). For the surface sample, the system delivered equivalent results in 33 hrs, a little less than half of the usual 72 hr for visual plate counting (Figure 13B). Similarly, for the air samples, the system saved over 1 day of the traditional 2 day testing time (Figure 13C). Note that by the end of the traditional testing period the Growth Direct System detected 24–55% more colonies than the visual plate counting method. This is because the digital imaging method detects microcolonies that are not yet visible by eye at the end of the traditional testing time. If the plates are incubated beyond the traditional testing time, these microcolonies become visible as well.

## Discussion

The data presented here demonstrate the potential to achieve rapid, accurate, sensitive, and non-destructive microbial enumeration by using automatic digital imaging to detect the autofluorescence of growing microbial cells. The Growth Direct System detects microbes representing diversity in phylogeny, metabolism, developmental program, and pigmentation. We have demonstrated that the fully automated system can achieve substantial time savings in key pharmaceutical quality control applications using samples from manufacturing facilities.

Our data show that digital imaging of cellular autofluorescence detects growing colonies much earlier than the traditional visual plate counting method. For example, the method detects *E. coli* colonies when they contain only about 120 cells, while the same colonies are not detected by the traditional visual method until they contain about 5×10^6^ cells. The average time to detection for *E. coli* using the autofluorescent method was 3.1 hr compared to an average of 8.5 hr for the visual plate counting method. The system detected microcolonies of the yeast, *C. albicans*, when they contained only about 12 cells, while visible detection requires colonies containing about 2×10^5^ cells. The average time to detection for *C. albicans* using the rapid method was 2.2 hr compared to 22 hr for the visual plate counting method. For both of these microbes, the autofluorescent method first detects colonies about 14–15 generations before they become visible. In general, for microbes that are otherwise similar, the system saves more time for the strain that grows more slowly. We present a model that predicts the time to detection based on four microbiological parameters (cell volume, doubling time, pre-growth time, and number of cells/CFU).

The system's on-board analysis software accurately discriminates against inanimate fluorescent debris by requiring that colonies grow over at least two imaging time points. We showed that the method has a dynamic range for *E. coli* that is more than 10 times higher than the traditional visual method because it detects microscopic colonies. Large visible colonies begin to merge – causing loss of counting accuracy – at much lower plating densities than do the microscopic colonies detected by autofluorescence.

Because the Growth Direct System uses the same core methodology as visual plate counting, the new method applies to the same broad range of applications for which visual culture methods are currently used. Our data show that the system can achieve time savings measured in days for key quality control applications (filterable liquid, air, and surface samples). The studies demonstrate that the method can rapidly detect mixed populations of unidentified environmental microbes. Though most of the experiments detailed in this paper used TSA medium, the technology also works with other growth media including R2A and SDA (Sabouraud Dextrose Agar) with no changes required to the software algorithms or imaging system (data not shown).

Compatibility of the rapid microbial enumeration method with existing microbial identification methods requires the ability to grow visible colonies (which are pure microbial cultures) from microcolonies. We demonstrated that the rapid method is non-destructive. Thus, it allows the detected microcolonies to continue to grow into visible colonies which can be subsequently used for identification. Our data show that cells from representative microbial groups are not killed even when exposed to much more blue light than is actually used in the system. The ability of the cells to survive exposure that is 10 times the level delivered by the system provides confidence that the method is unlikely to kill environmental microbes. We also showed that heating by the system does not significantly affect recovery of a heat-sensitive microbe, *P. fluorescens*. Thus, we have demonstrated that illumination and processing by the Growth Direct System is non-destructive to microbes. Some other devices and methods for high intensity illumination have, in contrast, caused a decrease in microbial recovery [16], [17]. Besides the differences in instrumentation and spectral regime, the lowered microbial recovery in some studies may be an artifact of photonic heating of the growth surface – an effect that we were careful to control for in our work.

New rapid methods for microbial enumeration have been widely implemented in the healthcare and food industries. In contrast, rapid testing has yet to gain a substantial foothold in pharmaceutical and personal care product industries. In these industries visual plate counting is used to detect microbial contamination in raw materials, in-process and finished products, and the manufacturing environment. Faster detection of contamination would enable manufacturers to more quickly release finished product, recognize that a contamination problem exists, and minimize the amount of discarded product. For these reasons regulators, manufacturers, trade groups, and quality assurance professionals in the pharmaceutical manufacturing industry have developed initiatives and guidelines to facilitate adoption of rapid microbial testing [15], [18], [19].

However, despite the high costs incurred by slow microbial testing and the availability of methods for faster testing [20], no rapid microbial enumeration technology has displaced a significant fraction of the more than 350 million traditional culture tests that are carried out by manufacturers of pharmaceuticals and personal care products each year [21]. Penetration of new rapid testing technologies in these industries has been impeded in part by failure to maintain some essential benefits of the current culture-based methods. Furthermore, demonstrating equivalence to regulatory reference tests can be problematic as most new methods are based on different principles and measure different quantities than the culture test gold standard.

Besides maintaining the key benefits of culture tests, the rapid method presented here should streamline regulatory validation since it is based on the same underlying principles and procedures as the regulatory gold standard. Manufacturers' validation effort should in large part be limited to the new automated aspects of the system. Demonstrating equivalence to the traditional method during validation is simplified because the new technology provides numerically equivalent results in the same units (CFU). In contrast, validation and demonstration of equivalence is expected to be more arduous for alternative rapid enumeration methods that are technically distinct from the current standard method and that deliver results using a different metric (for example, relative light units or number of cells with enzymatic activity).

We have shown that automated digital imaging of microcolony autofluorescence has the potential to address slow test turnaround and manual analysis, two of the major limitations of the visual plate counting method as practiced in industrial microbiology quality control. The system described here also offers advantages derived from its similarity to the current method such as compatibility with microbial identification, numerical equivalence, and congruence with the regulations. Compared to rapid methods that differ greatly from the traditional method, regulatory validation for the new method should be substantially streamlined. Manufacturers' validation efforts can focus simply on demonstrating that counting autofluorescent colonies using digital imaging is equivalent to visual colony counting.

Future publications will demonstrate the efficacy of the method in the context of a variety of key microbiological quality control applications.

## Materials and Methods

### Strains and Media and Membranes

All purchased strains were from the American Type Culture Collection (Manassas, VA), and include *E. coli* 8739, *B. diminuta* 11568, *C. albicans* 10231, and *P. fluorescens* 17397. Environmental isolates were identified by 16S ribosomal DNA sequencing [22]. Trypticase Soy Broth (TSB), Trypticase™ Soy Agar (TSA), and Standard Methods Agar were purchased from VWR International (West Chester, PA) or Northeast Laboratories (Winslow, ME). Phosphate buffered saline (PBS, 10 mM sodium phosphate pH 7.4, 140 mM NaCl, 3 mM KCl) was purchased from VWR. Membranes were 0.45 micron black mixed cellulose esters (MCE) membranes (Millipore Corp., Billerica, MA).

### Overview of the Instrumentation Functionality

The Growth Direct System (Rapid Micro Biosystems, Bedford MA) is an automated rapid microbial enumeration platform that integrates digital imaging, robotic handling, incubation, and software control. Bar coded Growth Cassettes are introduced to the system in a carrier. After being picked up by the robotic arm, the bar code is read and the cassette is imaged and then loaded into a built-in automated incubator. To build up an image time series, Growth Cassettes are periodically removed from the incubator, imaged, and returned to the incubator by the robotic system. Before imaging, a low-powered heater removes any condensation that may have formed on the optical cassette lid.

### Optical System

For imaging the cassette is held on a computer controlled stage with X, Y, and Z motion. Focusing is achieved using a non-contact laser distance sensor. The membrane in a Growth Cassette is illuminated with optically filtered columnated blue light (450–500 nm) from multiple light emitting diodes (LEDs). Incident light induces fluorescence in the target microcolonies which is collected by a high-collection efficiency, non-magnifying lens system. The emitted fluorescent light is transmitted through a green emission filter (510–560 nm) and collected by a charged-coupled device (CCD) digital camera. To analyze the entire working area of the membrane, the imager acquires nine contiguous image tiles. In early experiments, including the fluorescent images in figures 4 and 5, measurements of time-to-detection (Table 2), linearity (Figure 8) and precision, we used a prototype imager. The prototype had essentially the same functionality as the commercial system described above but was not fully automated and used an arc lamp rather than LEDs.

### Image Analysis

Onboard automated image analysis by custom software incorporates functions for background smoothing, object finding, and enumerating fluorescent objects that grow in size and intensity over time. Because the membrane and media display high-spatial-frequency optical noise which can vary with time, the software applies a background correction algorithm to smooth out the noise and separate signal from background. Clusters of neighboring pixels whose intensity significantly exceeds the local background are classified as objects. The software monitors key morphological parameters of the objects including their position, intensity, size, and other characteristics. Algorithms assess changes in these characteristics over time to identify the objects as either growing microcolonies or non-growing debris. Figure S4 demonstrates the accuracy of the automated image analysis software by comparing its detection of growing colonies to detection by visual inspection of images on a computer monitor. In the early experiments described in the previous section, we used prototype image analysis software and checked and edited the results by visual inspection of the images on a computer monitor.

### Growth Cassettes and Filtration Units

The body of the Growth Cassette (Rapid Micro Biosystems, Bedford MA) is custom made of injection molded styrenic plastics. The cassette incorporates a pouring lid that creates a flat agar surface, and a side-fill port for adding the agar (Figure S1). Just before use, the user removes the pouring lid, places a membrane on the agar surface, and then covers the cassette body with a sterile, clear lid that enables imaging. Filtration units (Rapid Micro Biosystems, Bedford MA) comprising plastic funnels and mixed cellulose ester membranes were used to filter liquid samples. To maintain membrane flatness during filtration, the membrane is supported by a porous plastic disk (Porex Corporation, Fairburn, GA). Units are sterilized by ethylene oxide treatment. In early experiments using the prototype imaging system, we used prototype cassettes and funnels which had essentially the same functionality as those described above.

### Determining the Time-to-Detection

For time to detection experiments using model organisms, log phase cells were serially diluted into PBS so that the final dilution (20 ml) contained approximately 100 CFU. *E. coli*, *B. diminuta*, and *C. albicans* were imaged at regular intervals (20 minutes for *E. coli* and 1 hour for *C. albicans* and *B. diminuta*). The first time point at which a colony can be found above the background of the image is its Growth Direct time to detection. We defined the time to detection for visible plate counting as the first time point at which a colony reaches a diameter of ≥0.5 mm. The median time to detection was calculated using a Probit analysis [23] of the proportion of colonies detected vs. time (See Figure S9). Doubling times were calculated using total pixel intensity of an imaged microcolony over time.

### Spectral Analysis of Microbial Autofluorescence

Cells from the species listed in Table 1 were grown in a thin confluent layer on membranes placed on TSA. Uninoculated controls were prepared and incubated in parallel for background measurements. The cassettes were mounted in a custom solid sample holder in a Fluorolog 3–22 spectrofluorometer (Horiba Jobin Yvon, Edison, NJ). The collection mirrors were set to the front facing setting, and the samples were scanned at 450 to 600 nm for emission (3 nm slit widths, integration times of 0.2 seconds at 5 nm increments) and from 400 to 550 nm for excitation (3 nm slit width, 10 nm increments). Data was collected in triplicate for each species.

Data from the three replicates were averaged. A plot combining the signal to background information for all the species was created by normalizing the values for each individual species to the median fluorescence value and then averaging the normalized values. See supplementary information for further explanation (Figure S10).

### Assessing Non-Destructive Detection

To test whether illumination by blue light is non-destructive to microbes, we exposed test strains to levels of light equivalent to 10 times that which they would be exposed to during normal operation. About 200 CFU were placed on TSA Growth Cassettes (without lids), inserted into an imager and exposed to light (1 W/cm^2^, maximum intensity) for 25 sec. The number of cells per CFU was determined by microscopic examination of at least 100 CFU prior to filtration. To isolate the effect of high light intensity from thermal effects, we took the following precautions to eliminate excessive heating due to the long exposures. Samples were illuminated in two consecutive 12.5 sec cycles and, for each cycle, the membranes were placed on a fresh pre-cooled (4°C) Growth Cassette. The maximum temperature reached transiently by the membranes was about 45°C, as measured in parallel experiments using a digital thermal probe (VWR). A set of control membranes was prepared at the same time and treated the same way except that the control membranes were not exposed to blue light. The Growth Cassettes were incubated overnight at 32.5°C.

Two approaches were used to demonstrate that system heating, which is a consequence of the imaging and condensation removal processes, does not kill *P. fluorescens*, a thermally sensitive microbe that does not grow at 42°C. Figure S6 demonstrates the heat sensitivity of *P. fluorescens*. Membranes containing *P. fluorescens* were placed on cassettes pre-warmed to 42°C – which is 9.5°C warmer than the cassettes in normal operation – and exposed to light equivalent to that in the normal imaging cycle. Plates were then incubated at 32.5°C alongside controls that did not experience temperatures above 32.5°C. For the second method we tested the survival of the same heat sensitive strain when cycled through the Growth Direct System at twice the frequency of imaging cycles compared to normal operation. For these experiments cassettes were incubated at 32.5°C and processed by the system (including condensation removal and imaging) every 1.5 hr. Control plates were incubated in parallel without processing.

### Determining Statistical Equivalence of Microbial Recovery with and without Stress

Our criteria for statistical equivalence in the heat and light stress experiments required that the test values be statistically equal to or greater than the control values. We assessed the equivalence using the Two One Sided t-test (TOST; [24]). To be judged as statistically equivalent to the control, the 95% confidence range of the test value must fall between two predetermined limits, called theta values. Theta values represent the cut-offs at which the two values are considered functionally equivalent. In practice, in pharmaceutical manufacturing, the test method must generally recover at least 70% of the control method. For the recovery of organisms in the stress experiments, we applied a stricter standard of “at least 90%”, hence we chose the theta value of −10% as our lower limit. We did not impose an upper limit for equivalency since higher recovery using the test method would be acceptable.

### Determining the Autofluorescent Intensity of Cells

Intrinsic microbial cellular fluorescence was determined using two methods: first using lawns of cells filtered onto membranes and measured with the Growth Direct System and second using flow cytometry [25]. For the lawn method, cells were grown to early log phase (<10^7^ CFU/ml) and washed in cold PBS. In triplicate, log phase cells were filtered such that approximately 3×10^7^ (*C. albicans*), 8×10^8^ (*E. coli*), or 7×10^9^ (*B. diminuta*) CFU were filtered onto a membrane. The membranes were rinsed twice (PBS, 50ml) and then mounted on Growth Cassettes (TSA) and imaged in the Growth Direct System. Cell concentrations were determined by plate counting (*E. coli*, *B. cepacia*) or using a hemocytometer (*C. albicans*). Negative control membranes were prepared using uninoculated TSB. To standardize fluorescence, a known quantity of beads calibrated in fluorescein equivalents (Bangs Laboratories, Fishers, IN) were filtered in lieu of bacteria. The autofluorescence was proportional to the number of cells on the membrane and that there was no “shadowing” effect from piling of microbial cells (data not shown).

For flow cytometry, cells were grown to early log phase (<10^7^ CFU/ml) and washed in cold PBS. Intrinsic fluorescence of individual cells was measured in a flow cytometer (CyFlow space, Partec, Mt. Laurel, NJ) with the adjustable laser set at 120 mW. Custom-labeled 1.8 µm fluorescent beads from Bangs Laboratories (Fishers, IN) were used to create a standard curve ranging from 198 to 1965 fluorescein equivalents for most species or 908 to 3642 for *C. albicans* [26], [27]. The cells were also examined microscopically after the wash steps to determine the distribution of cells/CFU.

### Determining the Number of Cells in Colonies

The number of cells in microcolonies as a function of incubation time was determined using two methods: scanning electron microscopy (SEM) and microbiological determination of the number of cells washed off of membranes containing known numbers of microcolonies. We used SEM to count the cells in microcolonies at various incubation times following the plating of log phase cells. We then used the measured doubling time to calculate, by extrapolation, the number of cells in microcolonies at the time-to-detection. Microcolonies were prepared for scanning electron microscopy (SEM) as follows. Log phase cells (>5000 CFU) were filtered and transferred to pre-warmed TSA plates. *E. coli* and *B. diminuta* were incubated at 35°C, while *C. albicans* was incubated at 25°C. At regular intervals (30 min for *E. coli*, 1 hour for *C. albicans* and *B. diminuta*) the membranes were transferred to filter paper (#3, Whatman International, Piscataway, NJ) soaked in a fixative (4% formaldehyde/2.5% glutaraldehyde/0.5× PBS at 4°C) and incubated overnight at 4°C. The membranes were then taken through an ethanol dehydration series (once each on 30% and 50% ethanol followed by twice on 70% ethanol) by serial transfer to filter paper soaked in the solvents followed by a final transfer to chloroform. The chloroform was evaporated and the membranes were prepared for SEM by coating with a 2 nm carbon film in a vacuum evaporator, followed by sputter coating with palladium and gold (10 nm) [28]. The membranes were then scanned in the SEM at about 2000× magnification to locate colonies, and counted at about 5000× magnification. To minimize undercounting of cells due to piling into layers, we only analyzed time points at which microcolonies averaged fewer than 15 cells. This corresponds to incubation times of 2 h for *E. coli*, 2 h for *C. albicans*, and 4 h for *B. diminuta*.

For analysis of the number of cells in microcolonies that had been washed off of membranes we prepared membranes with ∼5000 CFU. At various time points membranes were removed from the petri dishes and placed into 50 ml centrifuge tubes containing PBS (25 ml). Samples were agitated (Vortex Genie; Fisher Scientific, Waltham, MA; setting 8, 2 min) to remove cells from the membrane. Serial dilutions were prepared to determine the number of cells washed off at each time point, by either filtration or spread plate (in triplicate). We corrected for the efficiency with which cells were washed off of membranes (90% for *E. coli* and 98% for *C. albicans*, data not shown). We also corrected for the number of cells per CFU in the washed off material, which we determined by microscopic examination (average of 1.9 cells/CFU for *C. albicans*; ∼1 cell/CFU for *E. coli*).

We used essentially the same wash-off procedures for analyzing the number of cells in visible colonies, but with the following modifications. About 50 cells were filtered per membrane and incubated on TSA plates until the colonies were about 0.5 mm in diameter. To estimate the number of cells that remained on the membrane following washing, we stained the membranes with propidium iodide and examined them by fluorescence microscopy. The number of remaining cells did not exceed 6% of the cells for any of the species.

### Determining Cellular Volume

Cell volume estimates were made from shaken suspension-culture-grown log-phase cells in TSB. A drop of fresh cells was mounted on a microscope slide and covered with a standard cover slip. Light microscope images (400×, phase contrast) were captured by a CCD camera. The images were calibrated by imaging a 1 mm ruler (10 microns per tick mark) using the same microscope optics and camera. Images of the ruler and the cells were measured using ImagePro Plus software (Media Cybernetics, Bethesda, MD). The volume calculations for *E. coli* and *B. diminuta* were modeled after cylinders (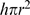). *C. albicans* was modeled as an ellipsoid with two distinct radii (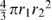). At least 50 cells were measured from each species.

### Testing Air, Surface, and Water Samples

All samples were collected in pharmaceutical manufacturing facilities. Air samples (500 liters) were taken using an Air Ideal™ sampler (bioMérieux SA, Marcy l'Etoile, France). Membranes were placed on the center of 55 mm contact plates (TSA) and placed in the air sampler. Following sampling, the membranes were transferred to a Growth Cassette and introduced to the Growth Direct System. For surface sampling, membranes were placed on the center of 55 mm contact plates (TSA) and the membrane edges were covered with polyfoil (Bel Art Products, Pequannock, NJ) to retain the membrane on the contact plate surface. Membranes were contacted with surfaces, the polyfoil was discarded, and the membranes were transferred to Growth Cassettes. Air samples were incubated at 32.5°C and imaged every 3 hours for 72 hrs. Surface samples were incubated at 32.5°C and imaged every 3 hours for 48 hrs. Water samples were inoculated into PBS, filtered onto membranes, and transferred to Growth Cassettes containing Standard Methods agar. Water samples were incubated at 32.5°C, and imaged every 3 hours for 72 hrs. Colonies were counted by eye at the end of the testing period.

## Supporting Information

Table S1 List of representative microbes that have been detected by the Growth Direct System. The Growth Direct System detects autofluorescent microcolonies from a broad range of strains as demonstrated by the breadth of the representative microbes listed in the table. All strains listed were identified by 16S ribosomal DNA sequencing or were purchased from a culture collection such as the ATCC. The Growth Direct System has also detected autofluorescent microcolonies corresponding to each of the many thousands of unidentified visible colonies isolated from environmental samples.
(0.02 MB PDF)

Table S2 Doubling times and lag phase of model organisms. Growth of cells in liquid media was monitored by titering samples of the growing cultures (in triplicate) at specific time intervals (20 minutes for *E. coli* and 1 hour for *C. albicans* and *B. diminuta*). For cells on membranes, the doubling time was determined based on the increase in microcolony fluorescence intensity over time. At least 10 colonies were analyzed for each species. The lag phase on membranes was determined from the wash-off experiment described in the “Determining the number of cells in colonies” section of the Materials and Methods.
(0.01 MB PDF)

Table S3 Rapid detection of bacteria that have been subjected to stress and injury. Stressed and injured microbes are commonplace in manufacturing environments. The table shows the results of experiments in which we determined how stress and injury typical of those that might be experienced by environmental water microbes affected the time to results for the common water-borne microbe *Ralstonia pickettii*. We compared the time to results for digital detection of autofluorescent microcolonies and for traditional visual plate counting. In other related work we have demonstrated the ability of the automated method to deliver rapid detection of cells that have been injured by heat and disinfectant treatment (data not shown). Note that in all such experiments, all microcolonies detected by the Growth Direct System gave rise to visible colonies upon further incubation. To simulate the nutritional stresses that might be experienced by water microbes, we subjected a strain of *R. pickettii* (that was isolated at a medical device company) to prolonged incubation (10 days) in pure water (water for injection) at 4°C or at room temperature (about 22°C). We also tested the injured *R. pickettii* cells that survived treatment with chlorinated city water (0.57 mg/L total chlorine; 1 hr, about 22°C) which killed 98.6% of the input microbes. After treatment with chlorinated water samples were neutralized using sodium thiosulfate (Standard methods for the examination of water and wastewater. 1998, Clesceri et al eds., Washington DC, Amer. Pub. Health Assoc.). As a non-stressed control sample we also tested log phase *R. pickettii* cells (TSB medium, 37°C, shaking). After plating, samples were analyzed using the Growth Direct System over the course of incubation (3 days; 35°C; R2A media; images taken at two hour intervals). For the rapid method colonies were counted at each interval using the system software. Colonies were scored as visually detectable when they reached 0.5 mm diameter. Table S3 compares the mean time to detection for *R. pickettii* cells for the rapid and traditional visual methods. The results show that, relative to the untreated log phase cells, prolonged growth in pure water and chlorine treatment increase the time to results either when detecting colonies by autofluorescence imaging or visually. The amount of delay in time to detection relative to the untreated log phase sample (*Δ_treated−logΦ_*) is greatest for sub-lethal injury by chlorine treatment followed by 10 day incubation in pure water at room temperature and 4°C. The table shows that the delay in the time to results (*Δ_treated−logΦ_*) is about the same for the rapid and traditional testing methods when cells were injured by chlorine (∼5 h) and cells incubated for 10 days in water at room temperature (∼4 h). When cells were incubated in water at 4°C for 10 days, the delay was slightly longer for detection by the rapid method (∼2 h compared to ∼1 h). As a consequence of the similarity in the treatment-induced delay for the two methods, the absolute time savings obtained using the rapid method are about the same whether the microbes are injured or not. This observation is consistent with the results of experiments on other strains that we injured using disinfectants and heat (data not shown). The results can be explained by the accepted model in which a period of physiologic adaptation and/or injury recovery is required before normal growth can occur following stress, change in chemical environment, or injury. According to this model and the one presented in the text, one would expect that stress and injury would extend the pre-growth period equivalently for both the rapid and traditional detection methods. In the absence of mutations that affect growth rate, the growth rates of cells should be the same once they adapt or recover and begin to grow. Thus, this model predicts that the time savings of the new method - which speeds detection by decreasing the number of generations of growth required - should be similar for samples with injured or healthy microbes.
(0.09 MB PDF)

Figure S1 The growth cassette design enables efficient optical imaging of microcolonies.
(0.05 MB PDF)

Figure S2 The Growth Direct System.
(0.06 MB PDF)

Figure S3 Key components of the Growth Direct System.
(0.09 MB PDF)

Figure S4 Accuracy of the Growth Direct System software. For water samples from a pharmaceutical plant, we compared the results obtained using the Growth Direct System image analysis software to analysis of the images by human visual inspection. The results plotted in the figure demonstrate that the automated image analysis software is comparable to human visual analysis. Note that human visual analysis is often considered the gold standard for this type of complex pattern recognition. For human visual analysis, the analyst found all of the objects in each image of the time series. Then, by comparing the objects on the various images in the time series, they determined which were growing objects and which were debris. Most of the microcolonies (607) became large enough to be visible to the human eye on the growth cassette by 48 hours. Some microcolonies (12) that can be detected in the Growth Direct System images do not become visible on the growth cassette by 48 hours. We detected visible colonies corresponding to each of the 12 microcolonies by examining the growth cassettes after further incubation Thus, all of the growing autofluorescent microcolonies that were detected by the Growth Direct System correspond to colonies that are detected by traditional visual inspection in this experiment.
(0.08 MB PDF)

Figure S5 The Growth Direct System software accurately detects colonies with diverse morphologies. Panel A shows the rapid detection of microcolonies of varying morphologies from a pharmaceutical plant environmental air sample. A photograph of the visible colonies seen at 72 hr (center) is surrounded by images of the corresponding microcolonies detected much earlier by the Growth Direct System. Panel B shows a mold microcolony as detected by the Growth Direct System image analysis software (red outline).
(0.08 MB PDF)

Figure S6 Temperature sensitivity of *P. fluorescens*. We chose *P. fluorescens*, a heat sensitive microbe, as a model to demonstrate that heating during processing by the Growth Direct System would not kill heat-sensitive environmental microbes. The ideal heat-sensitive test strain should be able to grow at 35°C (the standard “high” temperature for industrial microbiological work) but should grow poorly at higher temperatures. To show that *P. fluorescens* fits this profile, we tested its ability to grow at various temperatures. *P. fluorescens* was grown on Growth Direct System membranes mounted on Petri dishes (TSA) at a variety of temperatures ranging from 25°C to 42°C. The figure plots the two methods we used to assess temperature sensitivity. The bars show the number of colonies recovered after two days' growth at each temperature. These results demonstrate that there is no statistically significant difference in colony recovery for samples grown at temperatures from 25°C to 37°C (3 replicates, error bars indicate Standard Deviation). However, we recovered no colonies when samples were incubated at 42°C. We also measured the temperature dependence of colony size by analyzing digital images of the macroscopic (visible) colonies on the plates. The plot shows that there is no significant difference in the colony size for samples incubated at temperatures ranging from 25°C to 32.5°C. However, the colonies are significantly smaller when grown at 35°C and smaller yet when grown at 37°C. These data indicate that the growth of *P. fluorescens* slows significantly at temperatures above 32.5°C, and that it is incapable of growth at 42°C.
(0.07 MB PDF)

Figure S7 Extending dynamic range by measuring total fluorescence intensity of confluent microbial lawns. The plot demonstrates that the Growth Direct System has the potential to accurately enumerate sample microbial loads over a range of more than 6 orders of magnitude. This capability comes from combining two enumeration methods: one that is accurate in samples that give rise to distinct microcolonies (<7,800 CFU) and one that enumerates the total autofluorescence intensity in samples that give rise to confluent lawns of microbes. For samples with distinct microcolonies - those with less than 7,800 CFU - we used the prototype Growth Direct System image analysis software (Materials and Methods) to enumerate the microbes. For samples with more than 7,800 CFU, we compared the total image intensity to a standard curve developed in a separate experiment as described below. A standard curve for *E. coli* was created by measuring the total fluorescence intensity of standard samples containing large and defined numbers of bacteria. We measured the fluorescence intensity of the bacterial lawns that formed after growth for 4 hours. Log phase *E. coli* cells suspended in PBS (approximately 1×10^4^ to 1×10^8^ CFU) were filtered onto membranes and placed on pre-warmed Growth Cassettes (TSA). The concentration of bacteria in the standard samples was determined by plate counting analysis of 10 replicate dilutions in the countable range (30–300 CFU). The signal for each point on the standard curve was determined by subtracting the fluorescence intensity at the start of incubation from the intensity after incubation for 4 hrs. The figure shows the results of a blinded study comparing the Growth Direct System results to the results of the traditional plate counting method. One scientist generated a series of samples containing a range of bacteria starting with an *E. coli* culture in log phase. A second scientist, with no knowledge of the bacterial load in the various samples, prepared and analyzed the samples using the Growth Direct System and image analysis software. The second scientist examined the images taken after incubating for 4 hr, and those that appeared confluent or near confluent were analyzed by the total intensity of the filterable area as described above, and estimates for the count were derived from the standard curve. The remaining images were analyzed by the prototype enumeration software. In an automated implementation an algorithm could trigger the use of the high-level bioburden method based on a threshold of fluorescence intensity at a given time point.
(0.10 MB PDF)

Figure S8 The threshold number of cells required to first detect microcolonies is inversely proportional to the intrinsic cellular fluorescence. The data plots the number of cells required to first detect autofluorescent microcolonies (***N_thresh_***) vs the intrinsic cellular fluorescence (***F_cell_***) for *E. coli*, *B. diminuta*, and *C. albicans*. The slope of the best-fit line estimates (***F_thresh_***), the threshold level of fluorescence required to detect microcolonies using the Growth Direct System (Equation 1 in text) is inversely proportional to their intrinsic cellular fluorescence.
(0.07 MB PDF)

Figure S9 Determining the time to detection for model organisms. The plots show analysis of the time to detection for A) *C. albicans*, B) *E. coli*, and C) *B. diminuta*. Freshly grown cultures were filtered onto Growth Direct membranes, and then mounted onto growth cassettes. They were then imaged in the Growth Direct System over a number of closely spaced time intervals (see materials and methods for details). For each cassette, a different symbol was used to plot the percent of the total number of colonies vs. time. Using a Probit analysis on the combined data, the time to 50% detection was determined for each species.
(0.08 MB PDF)

Figure S10 The spectral dependence of the microbiological autofluorescence signal relative to the background signal from membranes saturated with growth medium. The figure uses a single microbial strain to illustrate the method we used to plot fluorescence signal to background for a large range of microbial strains. The fluorescent scans were performed on the surface of thin lawns of bacteria grown on filtration membranes mounted on media. An example of the raw data for a *B. cepacia* scan is shown in panel A. This plot should represent the signal from the *B. cepacia* cells plus the fluorescent background from the membrane. To measure the background, the same scan was performed on an un-inoculated membrane mounted on the same media, in this case TSA (panel B). To determine the signal-to-background ratio, we subtracted the values in panel B from those in panel A (whose results are shown in panel C) and then divided this difference by the values in panel B.
(0.07 MB PDF)
