# Supplementary material for: An Automated System for Rapid Non-Destructive Enumeration of Growing Microbes
Source: PLoS One. 2010 Jan 7;5(1):e8609. doi: 10.1371/journal.pone.0008609 (PMC2798718; doi:10.1371/journal.pone.0008609)
Supplement: Table S1 — List of representative microbes that have been detected by the Growth Direct System. The Growth Direct System detects autofluorescent microcolonies from a broad range of strains as demonstrated by the breadth of the representative microbes listed in the table. All strains listed were identified by 16S ribosomal DNA sequencing or were purchased from a culture collection such as the ATCC. The Growth Direct System has also detected autofluorescent microcolonies corresponding to each of the many thousands of unidentified visible colonies isolated from environmental samples. (0.02 MB PDF) [file pone.0008609.s001.pdf]

Table S1. List of representative microbes that have been detected by the Growth Direct System

Bacteria

Acidovorax delafieldii
Acidovorax sp.
Acidovorax temperans
Acinetobacter junii
Acholeplasma laidlawii
Afipia broomeae
Aquabacterium parvum
Arthrobacter sp.
Bacillus cereus
Bacillus clausii
Bacillus fusiformis
Bacillus gibsonii
Bacillus licheniformis
Bacillus megaterium
Bacillus pumilus
Bacillus sp.
Bacillus subtilis
Bacillus vortex
Bacteroides fragilis
Brachybacterium sp.
Bradyrhizobium spp.
Brevibacterium sp.
Brevundimonas diminuta
Burkholderia cepacia
Caulobacter leidyii
Chromobacterium violaceum
Clostridium sporogenes
Corynebacterium pseudodiphtheriticum
Corynebacterium sp.
Corynebacterium xerosis
Curtobacterium sp.
Deinococcus proteolyticus
Dermacoccus nishinomiyaensis
Enterobacter aerogenes
Enterococcus faecalis
Escherichia coli
Geobacillus stearothermophilus
Gluconacetobacter liquifaciens
Gluconacetobacter sacchari
Gluconobacter cerinus
Halomonas magadiensis
Hydrogenophagea sp.
Hyphomicrobium sp.
Kocuria kristinae
Kocuria rhizophila
Kytococcus sedentarius
Lactobacillus brevis
Lactobacillus delbrueckii
Lactobacillus lindneri
Macrococcus caseolyticus
Methylobacterium extorquens
Methylobacterium radiotolerans
Microbacterium luteolum
Microbacterium sp.
Micrococcus luteus
Moraxella osloensis
Mycobacterium chelonae
Mycoplasma arthritidis
Mycoplasma bovis
Mycoplasma fermentans
Mycoplasma hyorhinis
Mycoplasma orale
Mycoplasma pneumoniae
Mycoplasma synoviae
Myxococcus xanthus
Neisseria sp.
Paenibacillus lautus
Paenibacillus sp.
Pantoea agglomerans
Paracoccus sp.
Pectinatus frisingensis
Pediococcus damnosus
Porphyromonas gingivalis
Prevotella melaninogenica
Propionibacterium acnes
Proteus vulgaris
Pseudomonas aeruginosa
Pseudomonas fluorescens
Pseudomonas putida
Pseudomonas stutzeri
Ralstonia isidiosa
Ralstonia pickettii
Rhodococcus erythropolis
Roseomonas gilardii
Roseomonas sp.
Salmonella enterica
Serratia marcesens
Sphingomonas paucimobilis
Sphingomonas parapaucimobilis
Sphingomonas spp.
Sphingomonas terrae
Staphylococcus aureus
Staphylococcus capitis
Staphylococcus epidermidis
Staphylococcus equorum
Staphylococcus haemolyticus
Staphylococcus hominis
Staphylococcus saccharolyticus
Staphylococcus sp.
Staphylococcus warneri
Stenotrophomonas maltophilia
Streptococcus sp.
Streptomyces chrysomallus complex
Streptomyces coelicolor
Streptomyces sp.
Vibrio natriegens

Yeasts and molds

Alternaria alternata
Alternaria geophila
Arthrinium sacchari
Aspergillus flavus
Aspergillus fumigatus
Aspergillus niger
Aspergillus sp.
Aspergillus versicolor
Aureobasidium pullulans
Candida albicans
Candida parapsilosis
Chaetomium globosum
Chaetomium indicum
Cladosporium herbarum
Dekkera anomala
Dekkera bruxellensis
Epicoccum nigrum
Exophiala salmonis
Exophiala sp.
Fusarium solani
Penicillium camemberti
Penicillium chrysogeneum
Penicillium corylophylum
Penicillium notatum
Penicillium roqueforti
Rhizopus oligosporus
Saccharomyces cerevisiae
Schizophyllum commune
Schizophyllum fasciatum
Schizosaccharomyces pombe
Sporidiobolus johnsonii
Sporotrichum pruinosum
Torulaspora delbrueckii
Trichoderma asperellum
Zygosaccharomyces rouxii

The Growth Direct System detects autofluorescent microcolonies from a broad range of strains as demonstrated by the breadth of the representative microbes listed in the table. All strains listed were identified by 16S ribosomal DNA sequencing or were purchased from a culture collection such as the ATCC. The Growth Direct System has also detected autofluorescent microcolonies corresponding to each of the many thousands of unidentified visible colonies isolated from environmental samples.
