# Supplementary material for: An Automated System for Rapid Non-Destructive Enumeration of Growing Microbes
Source: PLoS One. 2010 Jan 7;5(1):e8609. doi: 10.1371/journal.pone.0008609 (PMC2798718; doi:10.1371/journal.pone.0008609)
Supplement: Table S2 — Doubling times and lag phase of model organisms. Growth of cells in liquid media was monitored by titering samples of the growing cultures (in triplicate) at specific time intervals (20 minutes for E. coli and 1 hour for C. albicans and B. diminuta). For cells on membranes, the doubling time was determined based on the increase in microcolony fluorescence intensity over time. At least 10 colonies were analyzed for each species. The lag phase on membranes was determined from the wash-off experiment described in the “Determining the number of cells in colonies” section of the Materials and Methods. (0.01 MB PDF) [file pone.0008609.s002.pdf]

Table S2. Doubling times and lag phase of model organisms

	**broth**[1]	**membranes**	**lag phase on membranes**
C albicans	82 min	74 min	60 min
E. coli	21 min	20 min	40 min
B. diminuta	64 min	55 min	ND

[1] log phase doubling times

Growth of cells in liquid media was monitored by titering samples of the growing cultures (in triplicate) at specific time intervals (20 minutes for *E. coli* and 1 hour for *C. albicans* and *B. diminuta*). For cells on membranes, the doubling time was determined based on the increase in microcolony fluorescence intensity over time. At least 10 colonies were analyzed for each species. The lag phase on membranes was determined from the wash-off experiment described in the “Determining the number of cells in colonies” section of the Materials and Methods.
