# Supplementary material for: An Automated System for Rapid Non-Destructive Enumeration of Growing Microbes
Source: PLoS One. 2010 Jan 7;5(1):e8609. doi: 10.1371/journal.pone.0008609 (PMC2798718; doi:10.1371/journal.pone.0008609)
Supplement: Table S3 — Rapid detection of bacteria that have been subjected to stress and injury. Stressed and injured microbes are commonplace in manufacturing environments. The table shows the results of experiments in which we determined how stress and injury typical of those that might be experienced by environmental water microbes affected the time to results for the common water-borne microbe Ralstonia pickettii. We compared the time to results for digital detection of autofluorescent microcolonies and for traditional visual plate counting. In other related work we have demonstrated the ability of the automated method to deliver rapid detection of cells that have been injured by heat and disinfectant treatment (data not shown). Note that in all such experiments, all microcolonies detected by the Growth Direct System gave rise to visible colonies upon further incubation. To simulate the nutritional stresses that might be experienced by water microbes, we subjected a strain of R. pickettii (that was isolated at a medical device company) to prolonged incubation (10 days) in pure water (water for injection) at 4°C or at room temperature (about 22°C). We also tested the injured R. pickettii cells that survived treatment with chlorinated city water (0.57 mg/L total chlorine; 1 hr, about 22°C) which killed 98.6% of the input microbes. After treatment with chlorinated water samples were neutralized using sodium thiosulfate (Standard methods for the examination of water and wastewater. 1998, Clesceri et al eds., Washington DC, Amer. Pub. Health Assoc.). As a non-stressed control sample we also tested log phase R. pickettii cells (TSB medium, 37°C, shaking). After plating, samples were analyzed using the Growth Direct System over the course of incubation (3 days; 35°C; R2A media; images taken at two hour intervals). For the rapid method colonies were counted at each interval using the system software. Colonies were scored as visually detectable when they reached 0.5 mm diameter. Table S3 comp [file pone.0008609.s003.pdf]

Table S3. ***Rapid detection of bacteria that have been subjected to stress and injury***

	**Growth Direct System** (hr) (uncertainty)[1]		**visual plate count** (hr) (uncertainty)		**time savings** (hr) (uncertainty)
**sample or treatment**	mean time to results	$\Delta t_{treated - log\Phi}$	mean time to results	$\Delta t_{treated - log\Phi}$	$\Delta t_{visible - Growth\ Direct}$
log phase	11.4 (0.1)	0	20.4 (0.1)	0	9.1 (0.2)
water, 10 days, 4°C	13.6 (0.1)	2.3 (0.2)	21.2 (0.1)	0.8 (0.2)	7.6 (0.2)
water, 10 days, room temp	15.2 (0.2)	3.8 (0.3)	24.3 (0.2)	3.9 (0.2)	9.1 (0.3)
chlorine (99% kill)	16.4 (0.4)	5.1 (0.5)	26.0 (0.5)	5.6 (0.5)	9.6 (0.9)

[1]*uncertainties expressed as ± 1 SEM*

Stressed and injured microbes are commonplace in manufacturing environments. The table shows the results of experiments in which we determined how stress and injury typical of those that might be experienced by environmental water microbes affected the time to results for the common water-borne microbe *Ralstonia pickettii*. We compared the time to results for digital detection of autofluorescent microcolonies and for traditional visual plate counting. In other related work we have demonstrated the ability of the automated method to deliver rapid detection of cells that have been injured by heat and disinfectant treatment (data not shown). Note that in all such experiments, all microcolonies detected by the Growth Direct System gave rise to visible colonies upon further incubation.

To simulate the nutritional stresses that might be experienced by water microbes, we subjected a strain of *R. pickettii* (that was isolated at a medical device company) to prolonged incubation (10 days) in pure water (water for injection) at 4°C or at room temperature (about 22°C). We also tested the injured *R. pickettii* cells that survived treatment with chlorinated city water (0.57 mg/L total chlorine; 1 hr, about 22°C) which killed 98.6% of the input microbes. After treatment with chlorinated water samples were neutralized using sodium thiosulfate (Standard methods for the examination of water and wastewater. 1998, Clesceri et al eds., Washington DC, Amer. Pub. Health Assoc.). As a non-stressed control sample we also tested log phase *R. pickettii* cells (TSB medium, 37°C, shaking). After plating, samples were analyzed using the Growth Direct System over the course of incubation (3 days; 35°C; R2A media; images taken at two hour intervals). For the rapid method colonies were counted at each interval using the system software. Colonies were scored as visually detectable when they reached 0.5 mm diameter.

Table S3 compares the mean time to detection for *R. pickettii* cells for the rapid and traditional visual methods. The results show that, relative to the untreated log phase cells, prolonged growth in pure water and chlorine treatment increase the time to results either when detecting colonies by autofluorescence imaging or visually. The amount of delay in time to detection relative to the untreated log phase sample ($\Delta t_{treated - log\Phi}$) is greatest for sub-lethal injury by chlorine treatment followed by 10 day incubation in pure water at room temperature and 4°C. The table shows that the delay in the time to results ($\Delta t_{treated - log\Phi}$) is about the same for the rapid and traditional testing methods when cells were injured by chlorine (~5 h) and cells incubated for 10 days in water at room temperature (~4 h). When cells were incubated in water at 4°C for 10 days, the delay was slightly longer for detection by the rapid method (~2 h compared to ~1 h).

As a consequence of the similarity in the treatment-induced delay for the two methods, the absolute time savings obtained using the rapid method are about the same whether the microbes are injured or not. This observation is consistent with the results of experiments on other strains that we injured using disinfectants and heat (data not shown). The results can be explained by the accepted model in which a period of physiologic adaptation and/or injury recovery is required before normal growth can occur following stress, change in chemical environment, or injury. According to this model and the one presented in the text, one would expect that stress and injury would extend the pre-growth period equivalently for both the rapid and traditional detection methods. In the absence of mutations that affect growth rate, the growth rates of cells should be the same once they adapt or recover and begin to grow. Thus, this model predicts that the time savings of the new method – which speeds detection by decreasing the number of generations of growth required − should be similar for samples with injured or healthy microbes.
