# Supplementary figures and images for: An Automated System for Rapid Non-Destructive Enumeration of Growing Microbes
Source: PLoS One. 2010 Jan 7;5(1):e8609. doi: 10.1371/journal.pone.0008609 (PMC2798718; doi:10.1371/journal.pone.0008609)

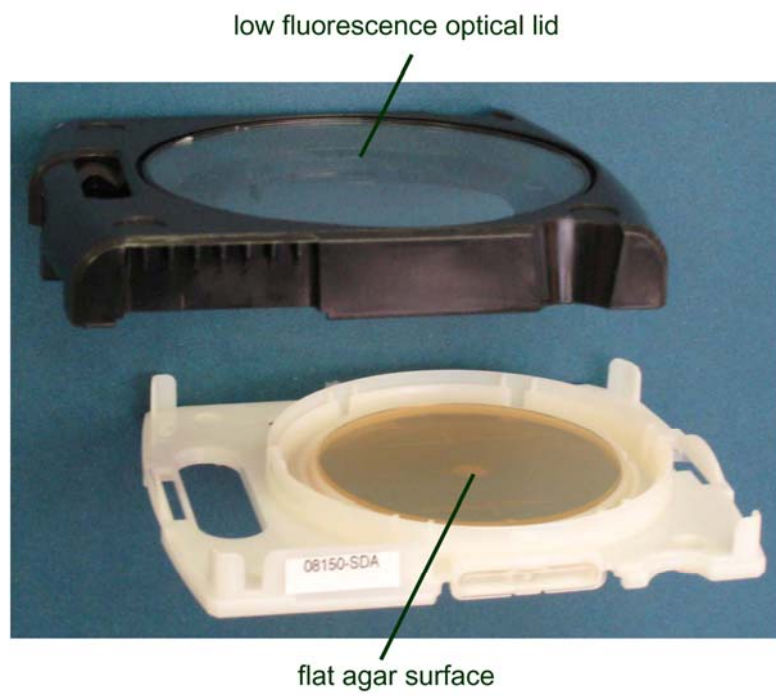

**Figure S1. The growth cassette design enables efficient optical imaging of microcolonies.**

Supplement: Figure S1 — The growth cassette design enables efficient optical imaging of microcolonies. (0.05 MB PDF) [file pone.0008609.s004.pdf]

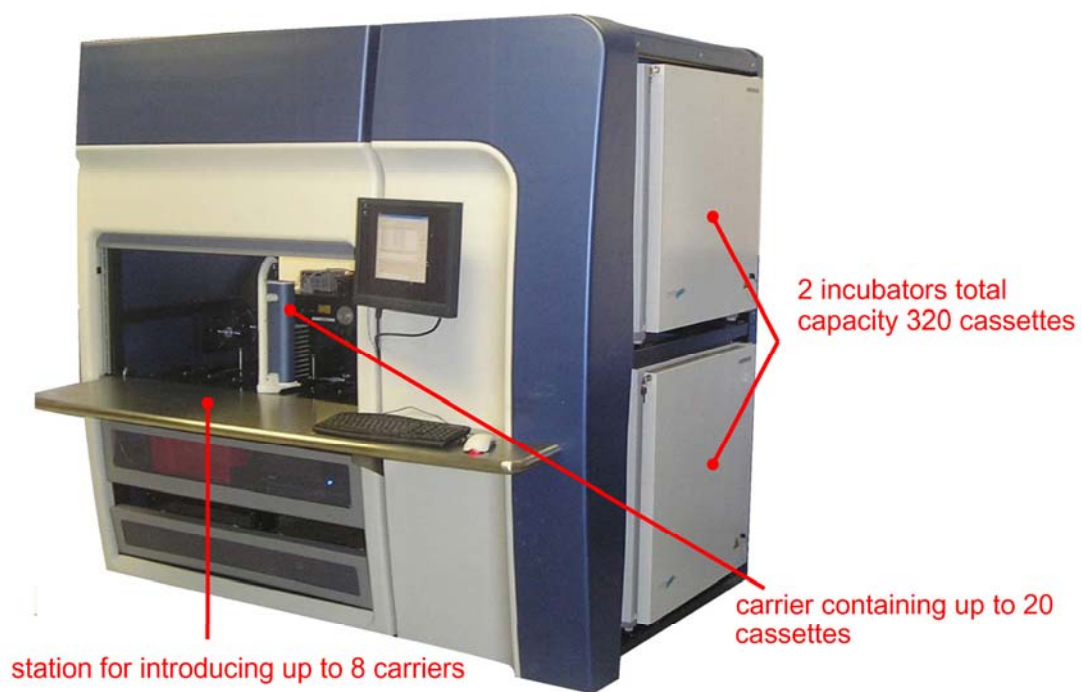

**Figure S2. The Growth Direct System.**

Supplement: Figure S2 — The Growth Direct System. (0.06 MB PDF) [file pone.0008609.s005.pdf]

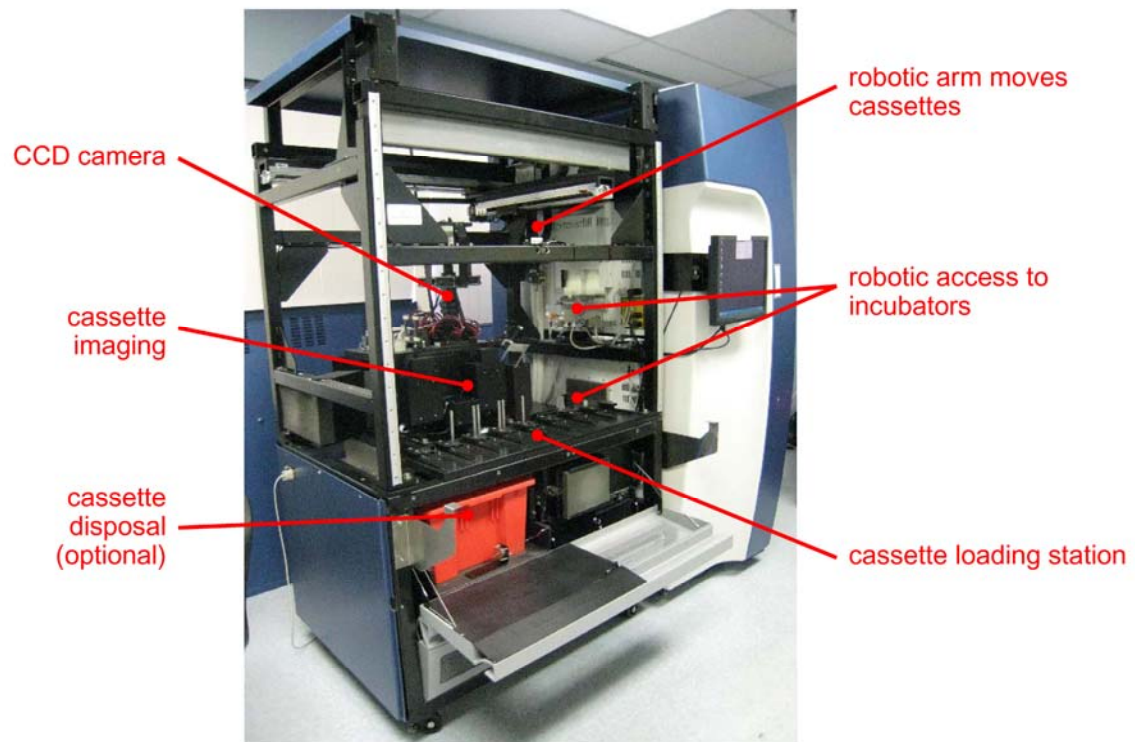

**Figure S3. Key components of the Growth Direct System.**

Supplement: Figure S3 — Key components of the Growth Direct System. (0.09 MB PDF) [file pone.0008609.s006.pdf]
