# Supplementary material for: An Automated System for Rapid Non-Destructive Enumeration of Growing Microbes
Source: PLoS One. 2010 Jan 7;5(1):e8609. doi: 10.1371/journal.pone.0008609 (PMC2798718; doi:10.1371/journal.pone.0008609)
Supplement: Figure S4 — Accuracy of the Growth Direct System software. For water samples from a pharmaceutical plant, we compared the results obtained using the Growth Direct System image analysis software to analysis of the images by human visual inspection. The results plotted in the figure demonstrate that the automated image analysis software is comparable to human visual analysis. Note that human visual analysis is often considered the gold standard for this type of complex pattern recognition. For human visual analysis, the analyst found all of the objects in each image of the time series. Then, by comparing the objects on the various images in the time series, they determined which were growing objects and which were debris. Most of the microcolonies (607) became large enough to be visible to the human eye on the growth cassette by 48 hours. Some microcolonies (12) that can be detected in the Growth Direct System images do not become visible on the growth cassette by 48 hours. We detected visible colonies corresponding to each of the 12 microcolonies by examining the growth cassettes after further incubation Thus, all of the growing autofluorescent microcolonies that were detected by the Growth Direct System correspond to colonies that are detected by traditional visual inspection in this experiment. (0.08 MB PDF) [file pone.0008609.s007.pdf]

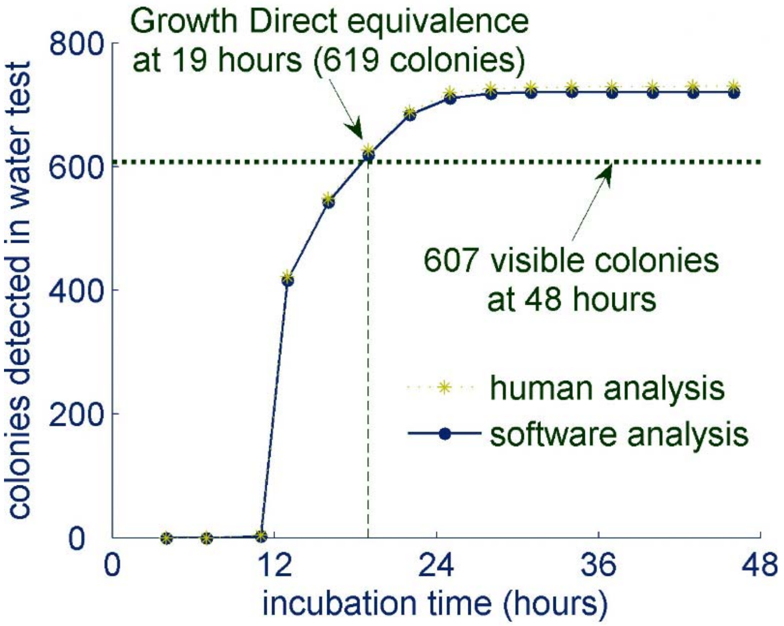

Figure S4. Accuracy of the Growth Direct System software. For water samples from a pharmaceutical plant, we compared the results obtained using the Growth Direct System image analysis software to analysis of the images by human visual inspection. The results plotted in the figure demonstrate that the automated image analysis software is comparable to human visual analysis. Note that human visual analysis is often considered the gold standard for this type of complex pattern recognition.

For human visual analysis, the analyst found all of the objects in each image of the time series. Then, by comparing the objects on the various images in the time series, they determined which were growing objects and which were debris. Most of the microcolonies (607) became large enough to be visible to the human eye on the growth cassette by 48 hours. Some microcolonies (12) that can be detected in the Growth Direct System images do not become visible on the growth cassette by 48 hours. We detected visible colonies corresponding to each of the 12 microcolonies by examining the growth cassettes after further incubation Thus, all of the growing autofluorescent microcolonies that were detected by the Growth Direct System correspond to colonies that are detected by traditional visual inspection in this experiment.
