# Supplementary material for: An Automated System for Rapid Non-Destructive Enumeration of Growing Microbes
Source: PLoS One. 2010 Jan 7;5(1):e8609. doi: 10.1371/journal.pone.0008609 (PMC2798718; doi:10.1371/journal.pone.0008609)
Supplement: Figure S5 — The Growth Direct System software accurately detects colonies with diverse morphologies. Panel A shows the rapid detection of microcolonies of varying morphologies from a pharmaceutical plant environmental air sample. A photograph of the visible colonies seen at 72 hr (center) is surrounded by images of the corresponding microcolonies detected much earlier by the Growth Direct System. Panel B shows a mold microcolony as detected by the Growth Direct System image analysis software (red outline). (0.08 MB PDF) [file pone.0008609.s008.pdf]

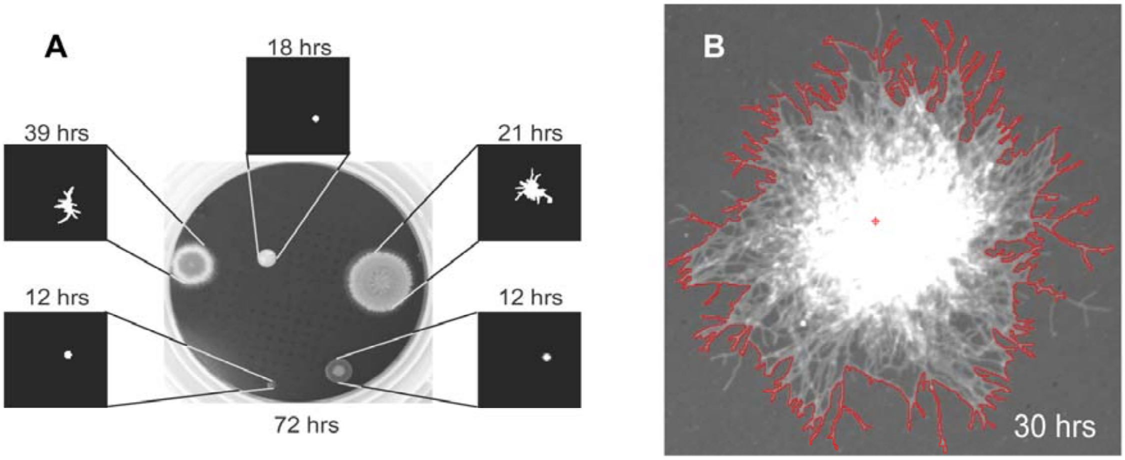

Figure S5. The Growth Direct System software accurately detects colonies with diverse morphologies. Panel A shows the rapid detection of microcolonies of varying morphologies from a pharmaceutical plant environmental air sample. A photograph of the visible colonies seen at 72 hr (center) is surrounded by images of the corresponding microcolonies detected much earlier by the Growth Direct System. Panel B shows a mold microcolony as detected by the Growth Direct System image analysis software (red outline).
