# Supplementary material for: An Automated System for Rapid Non-Destructive Enumeration of Growing Microbes
Source: PLoS One. 2010 Jan 7;5(1):e8609. doi: 10.1371/journal.pone.0008609 (PMC2798718; doi:10.1371/journal.pone.0008609)
Supplement: Figure S6 — Temperature sensitivity of P. fluorescens. We chose P. fluorescens, a heat sensitive microbe, as a model to demonstrate that heating during processing by the Growth Direct System would not kill heat-sensitive environmental microbes. The ideal heat-sensitive test strain should be able to grow at 35°C (the standard “high” temperature for industrial microbiological work) but should grow poorly at higher temperatures. To show that P. fluorescens fits this profile, we tested its ability to grow at various temperatures. P. fluorescens was grown on Growth Direct System membranes mounted on Petri dishes (TSA) at a variety of temperatures ranging from 25°C to 42°C. The figure plots the two methods we used to assess temperature sensitivity. The bars show the number of colonies recovered after two days' growth at each temperature. These results demonstrate that there is no statistically significant difference in colony recovery for samples grown at temperatures from 25°C to 37°C (3 replicates, error bars indicate Standard Deviation). However, we recovered no colonies when samples were incubated at 42°C. We also measured the temperature dependence of colony size by analyzing digital images of the macroscopic (visible) colonies on the plates. The plot shows that there is no significant difference in the colony size for samples incubated at temperatures ranging from 25°C to 32.5°C. However, the colonies are significantly smaller when grown at 35°C and smaller yet when grown at 37°C. These data indicate that the growth of P. fluorescens slows significantly at temperatures above 32.5°C, and that it is incapable of growth at 42°C. (0.07 MB PDF) [file pone.0008609.s009.pdf]

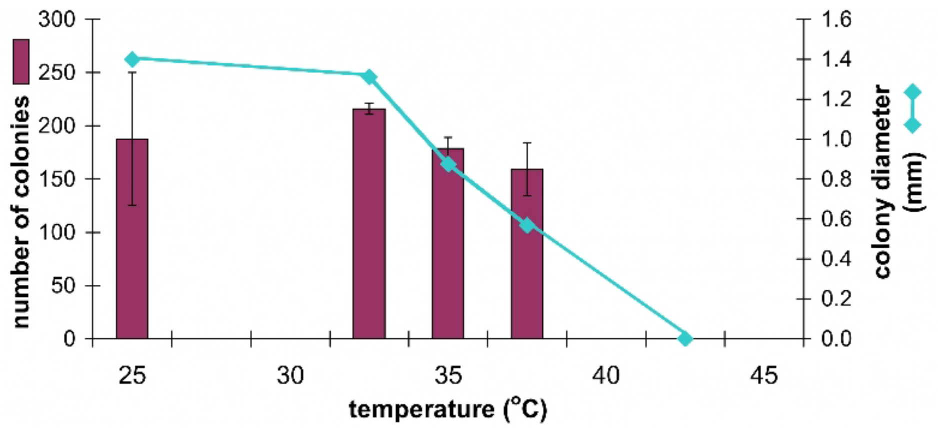

Figure S6. Temperature sensitivity of *P. fluorescens*. We chose *P. fluorescens*, a heat sensitive microbe, as a model to demonstrate that heating during processing by the Growth Direct System would not kill heat-sensitive environmental microbes. The ideal heat-sensitive test strain should be able to grow at 35°C (the standard “high” temperature for industrial microbiological work) but should grow poorly at higher temperatures. To show that *P. fluorescens* fits this profile, we tested its ability to grow at various temperatures. *P. fluorescens* was grown on Growth Direct System membranes mounted on Petri dishes (TSA) at a variety of temperatures ranging from 25°C to 42°C. The figure plots the two methods we used to assess temperature sensitivity. The bars show the number of colonies recovered after two days’ growth at each temperature. These results demonstrate that there is no statistically significant difference in colony recovery for samples grown at temperatures from 25°C to 37°C (3 replicates, error bars indicate Standard Deviation). However, we recovered no colonies when samples were incubated at 42°C. We also measured the temperature dependence of colony size by analyzing digital images of the macroscopic (visible) colonies on the plates. The plot shows that there is no significant difference in the colony size for samples incubated at temperatures ranging from 25°C to 32.5°C. However, the colonies are significantly smaller when grown at 35°C and smaller yet when grown at 37°C. These data indicate that the growth of *P. fluorescens* slows significantly at temperatures above 32.5°C, and that it is incapable of growth at 42°C.
