# Supplementary material for: An Automated System for Rapid Non-Destructive Enumeration of Growing Microbes
Source: PLoS One. 2010 Jan 7;5(1):e8609. doi: 10.1371/journal.pone.0008609 (PMC2798718; doi:10.1371/journal.pone.0008609)
Supplement: Figure S7 — Extending dynamic range by measuring total fluorescence intensity of confluent microbial lawns. The plot demonstrates that the Growth Direct System has the potential to accurately enumerate sample microbial loads over a range of more than 6 orders of magnitude. This capability comes from combining two enumeration methods: one that is accurate in samples that give rise to distinct microcolonies (<7,800 CFU) and one that enumerates the total autofluorescence intensity in samples that give rise to confluent lawns of microbes. For samples with distinct microcolonies - those with less than 7,800 CFU - we used the prototype Growth Direct System image analysis software (Materials and Methods) to enumerate the microbes. For samples with more than 7,800 CFU, we compared the total image intensity to a standard curve developed in a separate experiment as described below. A standard curve for E. coli was created by measuring the total fluorescence intensity of standard samples containing large and defined numbers of bacteria. We measured the fluorescence intensity of the bacterial lawns that formed after growth for 4 hours. Log phase E. coli cells suspended in PBS (approximately 1×10^4^ to 1×10^8^ CFU) were filtered onto membranes and placed on pre-warmed Growth Cassettes (TSA). The concentration of bacteria in the standard samples was determined by plate counting analysis of 10 replicate dilutions in the countable range (30–300 CFU). The signal for each point on the standard curve was determined by subtracting the fluorescence intensity at the start of incubation from the intensity after incubation for 4 hrs. The figure shows the results of a blinded study comparing the Growth Direct System results to the results of the traditional plate counting method. One scientist generated a series of samples containing a range of bacteria starting with an E. coli culture in log phase. A second scientist, with no knowledge of the bacterial load in the various samples, prepared and analyzed th [file pone.0008609.s010.pdf]

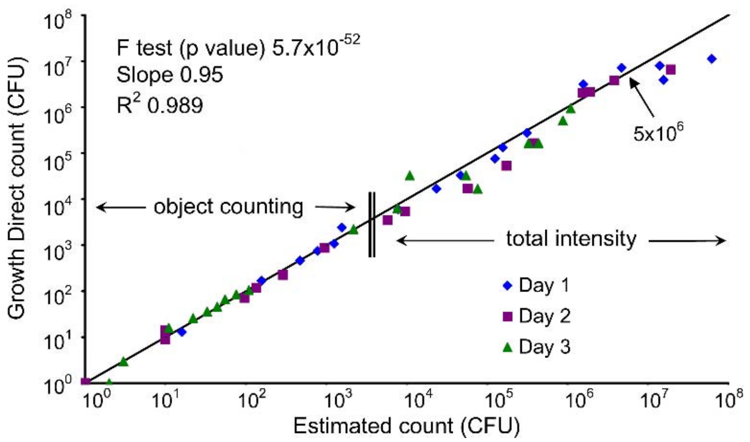

Figure S7. Extending dynamic range by measuring total fluorescence intensity of confluent microbial lawns. The plot demonstrates that the Growth Direct System has the potential to accurately enumerate sample microbial loads over a range of more than 6 orders of magnitude. This capability comes from combining two enumeration methods: one that is accurate in samples that give rise to distinct microcolonies (<7,800 CFU) and one that enumerates the total autofluorescence intensity in samples that give rise to confluent lawns of microbes.

For samples with distinct microcolonies − those with less than 7,800 CFU –we used the prototype Growth Direct System image analysis software (Materials and Methods) to enumerate the microbes. For samples with more than 7,800 CFU, we compared the total image intensity to a standard curve developed in a separate experiment as described below.

A standard curve for *E. coli* was created by measuring the total fluorescence intensity of standard samples containing large and defined numbers of bacteria. We measured the fluorescence intensity of the bacterial lawns that formed after growth for 4 hours. Log phase *E. coli* cells suspended in PBS (approximately $1X10^4$ to $1X10^8$ CFU) were filtered onto membranes and placed on pre-warmed Growth Cassettes (TSA). The concentration of bacteria in the standard samples was determined by plate counting analysis of 10 replicate dilutions in the countable range (30 – 300 CFU). The signal for each point on the standard curve was determined by subtracting the fluorescence intensity at the start of incubation from the intensity after incubation for 4 hrs.

The figure shows the results of a blinded study comparing the Growth Direct System results to the results of the traditional plate counting method. One scientist generated a series of samples containing a range of bacteria starting with an *E. coli* culture in log phase. A second scientist, with no knowledge of the bacterial load in the various samples, prepared and analyzed the samples using the Growth Direct System and image analysis software. The second scientist examined the images taken after incubating for 4 hr, and those that appeared confluent or near confluent were analyzed by the total intensity of the filterable area as described above, and estimates for the count were derived from the standard curve. The remaining images were analyzed by the prototype enumeration software. In an automated implementation an algorithm could trigger the use of the high-level bioburden method based on a threshold of fluorescence intensity at a given time point.
