# Supplementary material for: An Automated System for Rapid Non-Destructive Enumeration of Growing Microbes
Source: PLoS One. 2010 Jan 7;5(1):e8609. doi: 10.1371/journal.pone.0008609 (PMC2798718; doi:10.1371/journal.pone.0008609)
Supplement: Figure S8 — The threshold number of cells required to first detect microcolonies is inversely proportional to the intrinsic cellular fluorescence. The data plots the number of cells required to first detect autofluorescent microcolonies (Nthresh) vs the intrinsic cellular fluorescence (Fcell) for E. coli, B. diminuta, and C. albicans. The slope of the best-fit line estimates (Fthresh), the threshold level of fluorescence required to detect microcolonies using the Growth Direct System (Equation 1 in text) is inversely proportional to their intrinsic cellular fluorescence. (0.07 MB PDF) [file pone.0008609.s011.pdf]

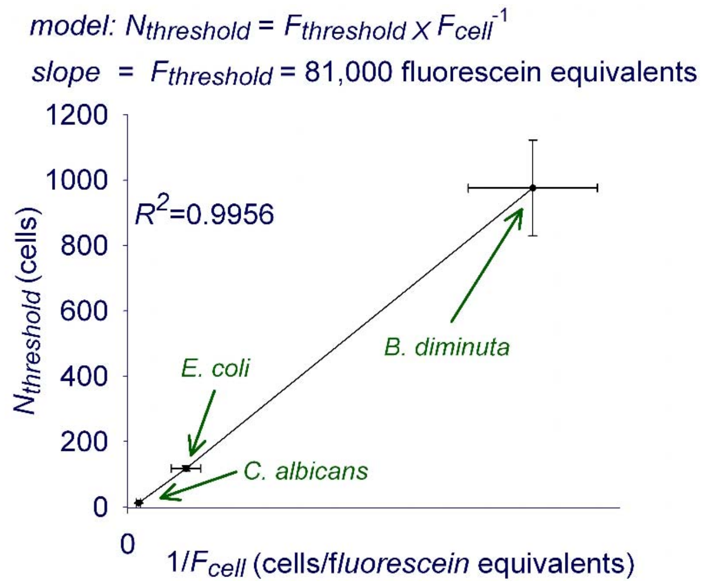

Figure S8. The threshold number of cells required to first detect microcolonies is inversely proportional to the intrinsic cellular fluorescence. The data plots the number of cells required to first detect autofluorescent microcolonies (N_{thresh}) vs the intrinsic cellular fluorescence (F_{cell}) for *E. coli*, *B. diminuta*, and *C. albicans*. The slope of the best-fit line estimates F_{thresh}, the threshold level of fluorescence required to detect microcolonies using the Growth Direct System (Equation 1 in text) is inversely proportional to their intrinsic cellular fluorescence.
