# Supplementary material for: An Automated System for Rapid Non-Destructive Enumeration of Growing Microbes
Source: PLoS One. 2010 Jan 7;5(1):e8609. doi: 10.1371/journal.pone.0008609 (PMC2798718; doi:10.1371/journal.pone.0008609)
Supplement: Figure S9 — Determining the time to detection for model organisms. The plots show analysis of the time to detection for A) C. albicans, B) E. coli, and C) B. diminuta. Freshly grown cultures were filtered onto Growth Direct membranes, and then mounted onto growth cassettes. They were then imaged in the Growth Direct System over a number of closely spaced time intervals (see materials and methods for details). For each cassette, a different symbol was used to plot the percent of the total number of colonies vs. time. Using a Probit analysis on the combined data, the time to 50% detection was determined for each species. (0.08 MB PDF) [file pone.0008609.s012.pdf]

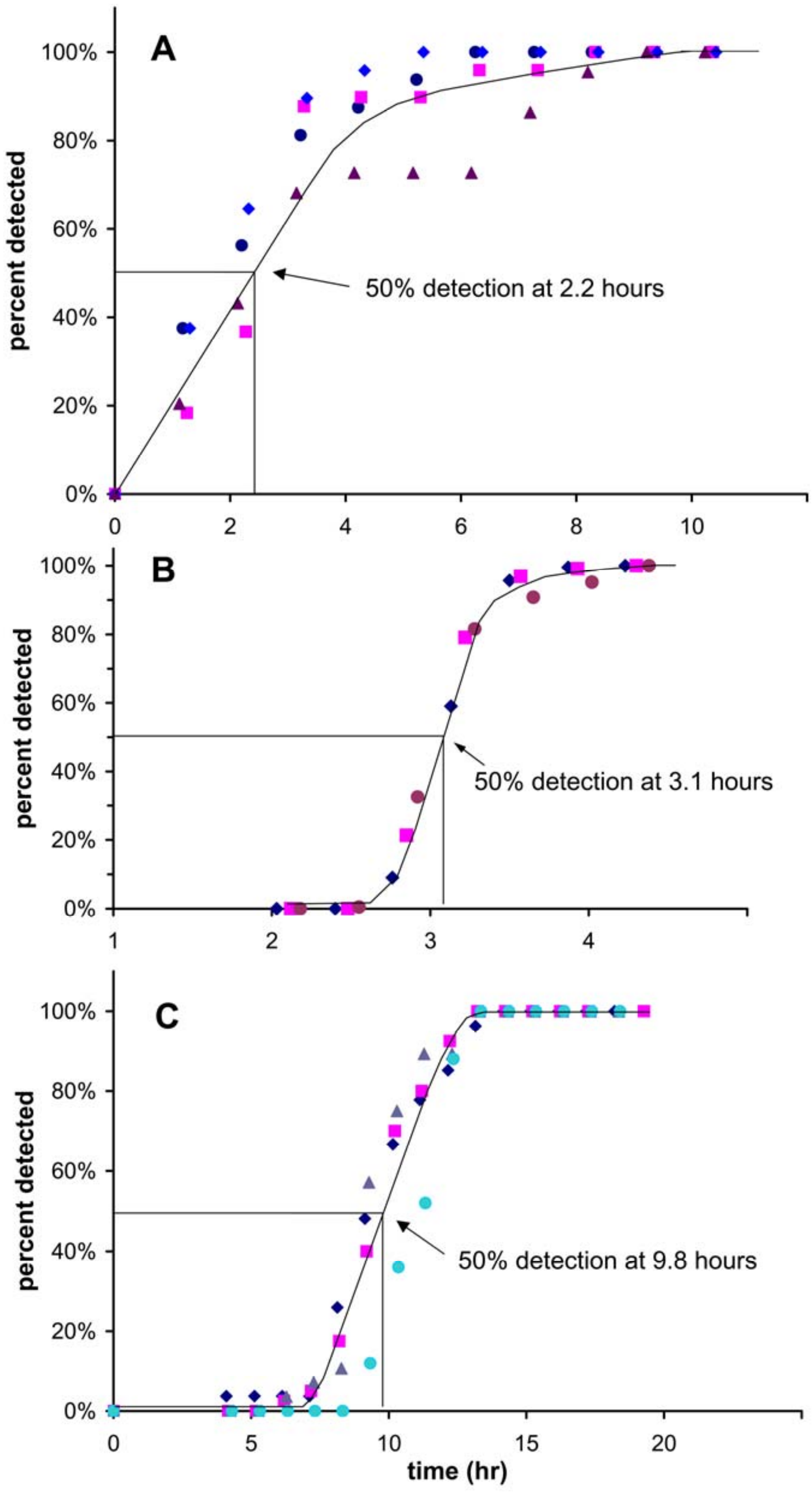

Figure S9. Determining the time to detection for model organisms. The plots show analysis of the time to detection for A) *C. albicans*, B) *E. coli*, and C) *B. diminuta.* Freshly grown cultures were filtered onto Growth Direct membranes, and then mounted onto growth cassettes. They were then imaged in the Growth Direct System over a number of closely spaced time intervals (see materials and methods for details). For each cassette, a different symbol was used to plot the percent of the total number of colonies vs. time. Using a Probit analysis on the combined data, the time to 50% detection was determined for each species.
