# Supplementary material for: An Automated System for Rapid Non-Destructive Enumeration of Growing Microbes
Source: PLoS One. 2010 Jan 7;5(1):e8609. doi: 10.1371/journal.pone.0008609 (PMC2798718; doi:10.1371/journal.pone.0008609)
Supplement: Figure S10 — The spectral dependence of the microbiological autofluorescence signal relative to the background signal from membranes saturated with growth medium. The figure uses a single microbial strain to illustrate the method we used to plot fluorescence signal to background for a large range of microbial strains. The fluorescent scans were performed on the surface of thin lawns of bacteria grown on filtration membranes mounted on media. An example of the raw data for a B. cepacia scan is shown in panel A. This plot should represent the signal from the B. cepacia cells plus the fluorescent background from the membrane. To measure the background, the same scan was performed on an un-inoculated membrane mounted on the same media, in this case TSA (panel B). To determine the signal-to-background ratio, we subtracted the values in panel B from those in panel A (whose results are shown in panel C) and then divided this difference by the values in panel B. (0.07 MB PDF) [file pone.0008609.s013.pdf]

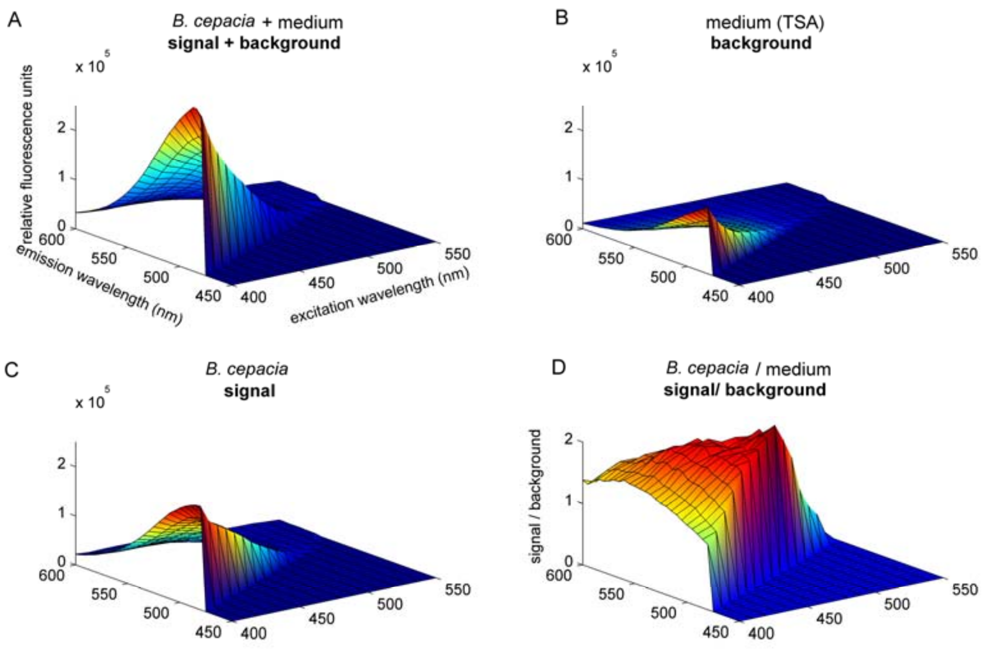

Figure S10. The spectral dependence of the microbiological autofluorescence signal relative to the background signal from membranes saturated with growth medium. The figure uses a single microbial strain to illustrate the method we used to plot fluorescence signal to background for a large range of microbial strains. The fluorescent scans were performed on the surface of thin lawns of bacteria grown on filtration membranes mounted on media. An example of the raw data for a *B. cepacia* scan is shown in panel A. This plot should represent the signal from the *B. cepacia* cells plus the fluorescent background from the membrane. To measure the background, the same scan was performed on an un-inoculated membrane mounted on the same media, in this case TSA (panel B). To determine the signal-to-background ratio, we subtracted the values in panel B from those in panel A (whose results are shown in panel C) and then divided this difference by the values in panel B.
